# Supplementary material for: Association between receiving the Aksi Bergizi Social Behavioral Change Communication (SBCC) intervention and dietary habits among secondary school students in Padang, Indonesia
Source: PLoS One. 2025 Sep 5;20(9):e0331312. doi: 10.1371/journal.pone.0331312 (PMC12412942; doi:10.1371/journal.pone.0331312)
Supplement: S5 File — Supporting Information – Questionnaire in Bilingual Format. (PDF) [file pone.0331312.s005.pdf]

KUESIONER PENELITIAN

Perilaku Kesehatan yang Dilaporkan Secara Mandiri dan Pendorong Perilaku pada Siswa Sekolah Menengah yang Mengikuti Program Promosi *Aksi Bergizi* di Sekolah Target dan Non-Target

Tanggal: Tanggal/Bulan/Tahun

Bagian A. Karakteristik demografi / Part A. Demographic characteristics

No	Pertanyaan	Question	Jawaban (Silahkan dicentang)	Answer (Please choose)
A2	Jenis kelamin	Sex	[ ] 1. Laki-laki<br>[ ] 2. Perempuan<br>[ ] 9. Menolak untuk menjawab	[ ] 1 Male<br>[ ] 2 Female<br>[ ] 9 Refuse to answer
A3	Tanggal lahir	Date of birth	Hari: [ ] [ ]<br>Bulan:[ ] [ ]<br>Tahun:[ ] [ ] [ ] [ ]	Day: [ ] [ ]<br>Month:[ ] [ ]<br>Year:[ ] [ ] [ ] [ ]
A4	Etnis	Ethnicity	[ ] 1. Minang<br>[ ] 2. Jawa<br>[ ] 3. Batak<br>[ ] 4. Sunda<br>[ ] 5. Lainnya<br>[ ] 88. Tidak tahu<br>[ ] 99. Menolak untuk menjawab	[ ] 1 Minangnese<br>[ ] 2 Javanese<br>[ ] 3 Bataknese<br>[ ] 4 Sundanese<br>[ ] 5 Others<br>[ ] 88 Don't know<br>[ ] 99 Refuse to answer
A5	Pekerjaan ayah	Fathers' occupation	[ ] 1. PNS<br>[ ] 2. Pegawai swasta<br>[ ] 3. Pedagang kecil<br>[ ] 4. Pemilik bisnis / pengusaha<br>[ ] 5. Buruh<br>[ ] 6. Pensiunan<br>[ ] 7. Bertani/ nelayan<br>[ ] 8. Profesional mandiri (pengacara, arsitek, lainnya)<br>[ ] 9. Tidak bekerja<br>[ ] 99. Menolak untuk menjawab	[ ] 1 Civil servant / state enterprise<br>[ ] 2 Private sector employee<br>[ ] 3 Small-scale vendors / service providers<br>[ ] 4 Business owner / entrepreneur<br>[ ] 5 Laborer / manual workers<br>[ ] 6 Retired / homemaker<br>[ ] 7 Agriculture / Fishery

			[ ] 0. Meninggal (***Lanjut ke pertanyaan A7***)	[ ] 8 Independent professions (e.g., lawyers, architects)<br>[ ] 9 Unemployed<br>[ ] 99 Refuse to answer<br>[ ] 0 Deceased ***(Skip to Question A7)***
A6	Fathers' education	Fathers' education	[ ] 1. Tidak pernah sekolah<br>[ ] 2. Tamat SD<br>[ ] 3. Tamat SMP<br>[ ] 4. Tamat SMA<br>[ ] 5. Tamat kejuruan<br>[ ] 6. Tamat D3<br>[ ] 7. Tamat D4<br>[ ] 8. Tamat S1<br>[ ] 9. Lebih tinggi dari S1<br>[ ] 88. Tidak yakin<br>[ ] 99. Menolak untuk menjawab	[ ] 1 Never went to school<br>[ ] 2 Primary school<br>[ ] 3 Junior high school<br>[ ] 4 High school<br>[ ] 5 Vocational certificate<br>[ ] 6 Associate's degree<br>[ ] 7 Vocational diploma<br>[ ] 8 Bachelor's degree<br>[ ] 9 Higher than bachelor's degree<br>[ ] 88 Not sure<br>[ ] 99 Refuse to answer
A7	Pekerjaan ibu	Mothers' occupation	[ ] 1. Ibu rumah tangga/ pensiunan<br>[ ] 2. PNS<br>[ ] 3. Pegawai swasta<br>[ ] 4. Pedagang kecil<br>[ ] 5. Pemilik bisnis / pengusaha<br>[ ] 6. Buruh<br>[ ] 7. Bertani/ nelayan<br>[ ] 8. Profesional mandiri (pengacara, arsitek, lainnya)<br>[ ] 9. Tidak bekerja<br>[ ] 99. Menolak untuk menjawab<br>[ ] 0. Meninggal (***Lanjut ke pertanyaan A9***)	[ ] 1 Civil servant / state enterprise<br>[ ] 2 Private sector employee<br>[ ] 3 Small-scale vendors / service providers<br>[ ] 4 Business owner / entrepreneur<br>[ ] 5 Laborer / manual workers<br>[ ] 6 Retired / homemaker<br>[ ] 7 Agriculture / Fishery<br>[ ] 8 Independent professions (e.g., lawyers, architects)<br>[ ] 9 Unemployed

				[ ] 99 Refuse to answer<br>[ ] 0 Deceased ***<u>(Skip to Question A9)</u>***
A8	Mothers' education	Mothers' education	[ ] 1. Tidak pernah sekolah<br>[ ] 2. Tamat SD<br>[ ] 3. Tamat SMP<br>[ ] 4. Tamat SMA<br>[ ] 5. Tamat kejuruan<br>[ ] 6. Tamat D3<br>[ ] 7. Tamat D4<br>[ ] 8. Tamat S1<br>[ ] 9. Lebih tinggi dari S1<br>[ ] 88. Tidak yakin<br>[ ] 99. Menolak untuk menjawab	[ ] 1 Never went to school<br>[ ] 2 Primary school<br>[ ] 3 Junior high school<br>[ ] 4 High school<br>[ ] 5 Vocational certificate<br>[ ] 6 Associate's degree<br>[ ] 7 Vocational diploma<br>[ ] 8 Bachelor's degree<br>[ ] 9 Higher than bachelor's degree<br>[ ] 88 Not sure<br>[ ] 99 Refuse to answer
A9	Pendapatan per bulan rumah tangga	Household Monthly Income	[ ] 1. < Rp. 1.000.000<br>[ ] 2. Rp. 1.000.000 hingga Rp. 2.000.000<br>[ ] 3. Rp. 2.000.001 hingga Rp. 3.000.000<br>[ ] 4. Rp. 3.000.001 hingga Rp. 4.000.000<br>[ ] 5. Rp. 4.000.001 hingga Rp. 5.000.000<br>[ ] 6. Rp. 5.000.001 hingga Rp. 6.000.000<br>[ ] 7. > Rp. 6.000.000<br>[ ] 8. Tidak yakin / tidak yakin / tidak tahu<br>[ ] 9. Menolak untuk menjawab	[ ] 1 No more than 1,000,000 IDR<br>[ ] 2 1,000,000 to 2,000,000 IDR<br>[ ] 3 2,000,001 to 3,000,000 IDR<br>[ ] 4 3,000,001 to 4,000,000 IDR<br>[ ] 5 4,000,001 to 5,000,000 IDR<br>[ ] 6 5,000,001 to 6,000,000 IDR<br>[ ] 7 More than 6,000,000 IDR<br>[ ] 88 Not sure / Uncertain / Don't know<br>[ ] 99 Refuse to answer

A13	Apa sumber media utama yang sering Anda temui untuk mendapatkan informasi tentang perilaku kesehatan?	What are the main media sources that you often encounter for obtaining information about health behaviors?	[ ] 1. Iklan televisi<br>[ ] 2. Acara di masyarakat<br>[ ] 3. Brosur informasi<br>[ ] 4. Keluarga dan/atau teman<br>[ ] 5. Platform media sosial (seperti: Facebook, Twitter, Instagram, dan lainnya)	[ ] 1 Television advertisements<br>[ ] 2 Community events<br>[ ] 3 Informational brochures<br>[ ] 4 Family and/or friends<br>[ ] 5 Social media platforms (e.g., Facebook, Twitter, Instagram, etc)

Bagian C. Pendorong perilaku untuk pola makan dan kesehatan / Part C. Dietary and health behavioral drivers

No	Pertanyaan	Question	Jawaban	Answer
		**Efikasi diri** / Self-Efficacy		
C20	Saya yakin bahwa saya dapat membatasi konsumsi minuman ber gula (misalnya, minuman bersoda, minuman energi) menjadi satu kali seminggu atau kurang.	I am confident that I can limit my consumption of sugary drinks (e.g., soda, energy drinks) to one per week or less	[ ] 1. Sangat tidak setuju<br>[ ] 2. Tidak setuju<br>[ ] 3. Setuju<br>[ ] 4. Sangat setuju	[ ] 1 Strongly disagree<br>[ ] 2 Disagree<br>[ ] 3 Agree<br>[ ] 4 Strongly agree
C21	Saya yakin bahwa saya dapat menolak godaan untuk terlibat dalam perilaku yang tidak sehat (misalnya, ngemil berlebihan, menghindari olahraga).	I am confident that I can resist the temptation to engage in unhealthy behaviors (e.g., excessive snacking, skipping exercise)	[ ] 1. Sangat tidak setuju<br>[ ] 2. Tidak setuju<br>[ ] 3. Setuju<br>[ ] 4. Sangat setuju	[ ] 1 Strongly disagree<br>[ ] 2 Disagree<br>[ ] 3 Agree<br>[ ] 4 Strongly agree

Bagian D. Kebiasaan diet / Part D. Dietary habits

No	Pertanyaan	Question	Jawaban (Silahkan dicentang)	Answer (Please choose)
catatan_ffq	**Kuesioner Frekuensi Konsumsi Makanan** Seberapa sering Anda makan makanan berikut?	**Food Consumption Frequency Questionnaire** How often do You eat the following foods?		
D1	Beras (beras putih)	Rice (white rice)	[ ] 1. Tidak pernah atau kurang dari satu kali per bulan<br>[ ] 2. 1-3 kali per bulan<br>[ ] 3. Satu kali per minggu<br>[ ] 4. 2-4 kali per minggu<br>[ ] 5. 5-6 kali per minggu<br>[ ] 6. 1 kali sehari<br>[ ] 7. Lebih dari satu kali sehari<br>[ ] 99. Menolak untuk menjawab	[ ] 1 Never or less than once per month<br>[ ] 2 1-3 times per month<br>[ ] 3 Once a week<br>[ ] 4 2-4 times per week<br>[ ] 5 5-6 times per week<br>[ ] 6 Once a day<br>[ ] 7 More than once a day<br>[ ] 99 Refuse to answer
D2	Produk gandum olahan (roti tawar, mie)	Refined wheat products (white bread, noodles)	[ ] 1. Tidak pernah atau kurang dari satu kali per bulan<br>[ ] 2. 1-3 kali per bulan<br>[ ] 3. Satu kali per minggu<br>[ ] 4. 2-4 kali per minggu<br>[ ] 5. 5-6 kali per minggu<br>[ ] 6. 1 kali sehari<br>[ ] 7. Lebih dari satu kali sehari<br>[ ] 99. Menolak	[ ] 1 Never or less than once per month<br>[ ] 2 1-3 times per month<br>[ ] 3 Once a week<br>[ ] 4 2-4 times per week<br>[ ] 5 5-6 times per week<br>[ ] 6 Once a day<br>[ ] 7 More than once a day<br>[ ] 99 Refuse to answer

No	Pertanyaan	Question	Jawaban (Silahkan dicentang)	Answer (Please choose)
			untuk menjawab	
D3	Beras merah	Coarse grain (brown rice)	[ ] 1. Tidak pernah atau kurang dari satu kali per bulan<br>[ ] 2. 1-3 kali per bulan<br>[ ] 3. Satu kali per minggu<br>[ ] 4. 2-4 kali per minggu<br>[ ] 5. 5-6 kali per minggu<br>[ ] 6. 1 kali sehari<br>[ ] 7. Lebih dari satu kali sehari<br>[ ] 99. Menolak untuk menjawab	[ ] 1 Never or less than once per month<br>[ ] 2 1-3 times per month<br>[ ] 3 Once a week<br>[ ] 4 2-4 times per week<br>[ ] 5 5-6 times per week<br>[ ] 6 Once a day<br>[ ] 7 More than once a day<br>[ ] 99 Refuse to answer
D4	Produk gandum utuh (misalnya, roti gandum, mie gandum)	Whole grain wheat products (e.g., brown bread, whole wheat noodles)	[ ] 1. Tidak pernah atau kurang dari satu kali per bulan<br>[ ] 2. 1-3 kali per bulan<br>[ ] 3. Satu kali per minggu<br>[ ] 4. 2-4 kali per minggu<br>[ ] 5. 5-6 kali per minggu<br>[ ] 6. 1 kali sehari<br>[ ] 7. Lebih dari satu kali sehari<br>[ ] 99. Menolak untuk menjawab	[ ] 1 Never or less than once per month<br>[ ] 2 1-3 times per month<br>[ ] 3 Once a week<br>[ ] 4 2-4 times per week<br>[ ] 5 5-6 times per week<br>[ ] 6 Once a day<br>[ ] 7 More than once a day<br>[ ] 99 Refuse to answer
D5	Umbi-umbian (singkong, talas, ubi jalar putih, kentang)	Tubers (cassava, taro, white yams, white potato)	[ ] 1. Tidak pernah atau kurang dari satu kali per bulan<br>[ ] 2. 1-3 kali per bulan<br>[ ] 3. Satu kali per minggu<br>[ ] 4. 2-4 kali per minggu	[ ] 1 Never or less than once per month<br>[ ] 2 1-3 times per month<br>[ ] 3 Once a week<br>[ ] 4 2-4 times per week<br>[ ] 5 5-6 times per week

No	Pertanyaan	Question	Jawaban (Silahkan dicentang)	Answer (Please choose)
			[ ] 5. 5-6 kali per minggu<br>[ ] 6. 1 kali sehari<br>[ ] 7. Lebih dari satu kali sehari<br>[ ] 99. Menolak untuk menjawab	[ ] 6 Once a day<br>[ ] 7 More than once a day<br>[ ] 99 Refuse to answer
D6	Daging (sapi, domba)	Meat (beef, mutton)	[ ] 1. Tidak pernah atau kurang dari satu kali per bulan<br>[ ] 2. 1-3 kali per bulan<br>[ ] 3. Satu kali per minggu<br>[ ] 4. 2-4 kali per minggu<br>[ ] 5. 5-6 kali per minggu<br>[ ] 6. 1 kali sehari<br>[ ] 7. Lebih dari satu kali sehari<br>[ ] 99. Menolak untuk menjawab	[ ] 1 Never or less than once per month<br>[ ] 2 1-3 times per month<br>[ ] 3 Once a week<br>[ ] 4 2-4 times per week<br>[ ] 5 5-6 times per week<br>[ ] 6 Once a day<br>[ ] 7 More than once a day<br>[ ] 99 Refuse to answer
D7	Produk ungags (bebek, ayam)	Poultry (duck, chicken)	[ ] 1. Tidak pernah atau kurang dari satu kali per bulan<br>[ ] 2. 1-3 kali per bulan<br>[ ] 3. Satu kali per minggu<br>[ ] 4. 2-4 kali per minggu<br>[ ] 5. 5-6 kali per minggu<br>[ ] 6. 1 kali sehari<br>[ ] 7. Lebih dari satu kali sehari<br>[ ] 99. Menolak untuk menjawab	[ ] 1 Never or less than once per month<br>[ ] 2 1-3 times per month<br>[ ] 3 Once a week<br>[ ] 4 2-4 times per week<br>[ ] 5 5-6 times per week<br>[ ] 6 Once a day<br>[ ] 7 More than once a day<br>[ ] 99 Refuse to answer
D8a	Ikan (mentah, panggang, sup, tidak digoreng)	Fish (raw, grilled, soup, not deep-fried)	[ ] 1. Tidak pernah atau kurang dari satu kali per bulan<br>[ ] 2. 1-3 kali per bulan	[ ] 1 Never or less than once per month<br>[ ] 2 1-3 times per month<br>[ ] 3 Once a week

No	Pertanyaan	Question	Jawaban (Silahkan dicentang)	Answer (Please choose)
			[ ] 3. Satu kali per minggu<br>[ ] 4. 2-4 kali per minggu<br>[ ] 5. 5-6 kali per minggu<br>[ ] 6. 1 kali sehari<br>[ ] 7. Lebih dari satu kali sehari<br>[ ] 99. Menolak untuk menjawab	[ ] 4 2-4 times per week<br>[ ] 5 5-6 times per week<br>[ ] 6 Once a day<br>[ ] 7 More than once a day<br>[ ] 99 Refuse to answer
D8b	Produk laut segar (misalnya, kerang, udang, kepiting, gurita)	Fresh seafood (e.g., clams, prawns, crabs, octopus)	[ ] 1. Tidak pernah atau kurang dari satu kali per bulan<br>[ ] 2. 1-3 kali per bulan<br>[ ] 3. Satu kali per minggu<br>[ ] 4. 2-4 kali per minggu<br>[ ] 5. 5-6 kali per minggu<br>[ ] 6. 1 kali sehari<br>[ ] 7. Lebih dari satu kali sehari<br>[ ] 99. Menolak untuk menjawab	[ ] 1 Never or less than once per month<br>[ ] 2 1-3 times per month<br>[ ] 3 Once a week<br>[ ] 4 2-4 times per week<br>[ ] 5 5-6 times per week<br>[ ] 6 Once a day<br>[ ] 7 More than once a day<br>[ ] 99 Refuse to answer
D9	Telur	Eggs	[ ] 1. Tidak pernah atau kurang dari satu kali per bulan<br>[ ] 2. 1-3 kali per bulan<br>[ ] 3. Satu kali per minggu<br>[ ] 4. 2-4 kali per minggu<br>[ ] 5. 5-6 kali per minggu<br>[ ] 6. 1 kali sehari<br>[ ] 7. Lebih dari satu kali sehari<br>[ ] 99. Menolak untuk menjawab	[ ] 1 Never or less than once per month<br>[ ] 2 1-3 times per month<br>[ ] 3 Once a week<br>[ ] 4 2-4 times per week<br>[ ] 5 5-6 times per week<br>[ ] 6 Once a day<br>[ ] 7 More than once a day<br>[ ] 99 Refuse to answer

No	Pertanyaan	Question	Jawaban (Silahkan dicentang)	Answer (Please choose)
D10	Sayuran berdaun hijau (misalnya, sawi hijau, kacang panjang, kale, bayam, sawi, timun)	Leafy green vegetables (e.g., Chinese cabbage, long bean, kale, spinach, yu choy, cucumber)	[ ] 1. Tidak pernah atau kurang dari satu kali per bulan<br>[ ] 2. 1-3 kali per bulan<br>[ ] 3. Satu kali per minggu<br>[ ] 4. 2-4 kali per minggu<br>[ ] 5. 5-6 kali per minggu<br>[ ] 6. 1 kali sehari<br>[ ] 7. Lebih dari satu kali sehari<br>[ ] 99. Menolak untuk menjawab	[ ] 1 Never or less than once per month<br>[ ] 2 1-3 times per month<br>[ ] 3 Once a week<br>[ ] 4 2-4 times per week<br>[ ] 5 5-6 times per week<br>[ ] 6 Once a day<br>[ ] 7 More than once a day<br>[ ] 99 Refuse to answer
D11	Sayuran berwarna kuning atau oranye (misalnya, labu, ubi jalar, wortel, pepaya matang)	Yellow or orange vegetables (e.g., pumpkin, sweet potatoes, carrots, ripened papaya)	[ ] 1. Tidak pernah atau kurang dari satu kali per bulan<br>[ ] 2. 1-3 kali per bulan<br>[ ] 3. Satu kali per minggu<br>[ ] 4. 2-4 kali per minggu<br>[ ] 5. 5-6 kali per minggu<br>[ ] 6. 1 kali sehari<br>[ ] 7. Lebih dari satu kali sehari<br>[ ] 99. Menolak untuk menjawab	[ ] 1 Never or less than once per month<br>[ ] 2 1-3 times per month<br>[ ] 3 Once a week<br>[ ] 4 2-4 times per week<br>[ ] 5 5-6 times per week<br>[ ] 6 Once a day<br>[ ] 7 More than once a day<br>[ ] 99 Refuse to answer
D12	Produk kedelai (misalnya., tahu, tempe)	Soybean products (e.g., tofu)	[ ] 1. Tidak pernah atau kurang dari satu kali per bulan<br>[ ] 2. 1-3 kali per bulan<br>[ ] 3. Satu kali per minggu<br>[ ] 4. 2-4 kali per minggu<br>[ ] 5. 5-6 kali per minggu<br>[ ] 6. 1 kali sehari<br>[ ] 7. Lebih dari	[ ] 1 Never or less than once per month<br>[ ] 2 1-3 times per month<br>[ ] 3 Once a week<br>[ ] 4 2-4 times per week<br>[ ] 5 5-6 times per week<br>[ ] 6 Once a day<br>[ ] 7 More than once a day<br>[ ] 99 Refuse to

No	Pertanyaan	Question	Jawaban (Silahkan dicentang)	Answer (Please choose)
			satu kali sehari<br>[ ] 99. Menolak untuk menjawab	answer
D13	Sayuran yang diawetkan (misalnya, sayuran kaleng)	Preserved vegetables (e.g., canned pickled vegetables)	[ ] 1. Tidak pernah atau kurang dari satu kali per bulan<br>[ ] 2. 1-3 kali per bulan<br>[ ] 3. Satu kali per minggu<br>[ ] 4. 2-4 kali per minggu<br>[ ] 5. 5-6 kali per minggu<br>[ ] 6. 1 kali sehari<br>[ ] 7. Lebih dari satu kali sehari<br>[ ] 99. Menolak untuk menjawab	[ ] 1 Never or less than once per month<br>[ ] 2 1-3 times per month<br>[ ] 3 Once a week<br>[ ] 4 2-4 times per week<br>[ ] 5 5-6 times per week<br>[ ] 6 Once a day<br>[ ] 7 More than once a day<br>[ ] 99 Refuse to answer
D14	Buah segar	Fresh fruits	[ ] 1. Tidak pernah atau kurang dari satu kali per bulan<br>[ ] 2. 1-3 kali per bulan<br>[ ] 3. Satu kali per minggu<br>[ ] 4. 2-4 kali per minggu<br>[ ] 5. 5-6 kali per minggu<br>[ ] 6. 1 kali sehari<br>[ ] 7. Lebih dari satu kali sehari<br>[ ] 99. Menolak untuk menjawab	[ ] 1 Never or less than once per month<br>[ ] 2 1-3 times per month<br>[ ] 3 Once a week<br>[ ] 4 2-4 times per week<br>[ ] 5 5-6 times per week<br>[ ] 6 Once a day<br>[ ] 7 More than once a day<br>[ ] 99 Refuse to answer
D15	Produk susu (susu segar, susu bubuk, susu kemasan)	Dairy products (fresh milk, powdered milk, boxed milk)	[ ] 1. Tidak pernah atau kurang dari satu kali per bulan<br>[ ] 2. 1-3 kali per bulan<br>[ ] 3. Satu kali per minggu<br>[ ] 4. 2-4 kali per minggu	[ ] 1 Never or less than once per month<br>[ ] 2 1-3 times per month<br>[ ] 3 Once a week<br>[ ] 4 2-4 times per week<br>[ ] 5 5-6 times per week

No	Pertanyaan	Question	Jawaban (Silahkan dicentang)	Answer (Please choose)
			[ ] 5. 5-6 kali per minggu<br>[ ] 6. 1 kali sehari<br>[ ] 7. Lebih dari satu kali sehari<br>[ ] 99. Menolak untuk menjawab	[ ] 6 Once a day<br>[ ] 7 More than once a day<br>[ ] 99 Refuse to answer
D16	Makanan ringan kemasan (misalnya, chiki jagung, keripik kentang Lay)	Packaged snacks (e.g., corn puffs, Lay potato chips)	[ ] 1. Tidak pernah atau kurang dari satu kali per bulan<br>[ ] 2. 1-3 kali per bulan<br>[ ] 3. Satu kali per minggu<br>[ ] 4. 2-4 kali per minggu<br>[ ] 5. 5-6 kali per minggu<br>[ ] 6. 1 kali sehari<br>[ ] 7. Lebih dari satu kali sehari<br>[ ] 99. Menolak untuk menjawab	[ ] 1 Never or less than once per month<br>[ ] 2 1-3 times per month<br>[ ] 3 Once a week<br>[ ] 4 2-4 times per week<br>[ ] 5 5-6 times per week<br>[ ] 6 Once a day<br>[ ] 7 More than once a day<br>[ ] 99 Refuse to answer
D17	Makanan ringan tanpa kemasan (donat, kentang goreng, roti, dll.)	Unpackaged snacks (donuts, french fries, roti, etc.)	[ ] 1. Tidak pernah atau kurang dari satu kali per bulan<br>[ ] 2. 1-3 kali per bulan<br>[ ] 3. Satu kali per minggu<br>[ ] 4. 2-4 kali per minggu<br>[ ] 5. 5-6 kali per minggu<br>[ ] 6. 1 kali sehari<br>[ ] 7. Lebih dari satu kali sehari<br>[ ] 99. Menolak untuk menjawab	[ ] 1 Never or less than once per month<br>[ ] 2 1-3 times per month<br>[ ] 3 Once a week<br>[ ] 4 2-4 times per week<br>[ ] 5 5-6 times per week<br>[ ] 6 Once a day<br>[ ] 7 More than once a day<br>[ ] 99 Refuse to answer
D18	Minuman atau yang diberi pemanis (susu	Sweetened drinks or condiments (soy milk, soft drinks, coke, coffee or tea with sugar,	[ ] 1. Tidak pernah atau kurang dari satu kali per bulan<br>[ ] 2. 1-3 kali per bulan	[ ] 1 Never or less than once per month<br>[ ] 2 1-3 times per month<br>[ ] 3 Once a week

No	Pertanyaan	Question	Jawaban (Silahkan dicentang)	Answer (Please choose)
	kedelai, minuman ringan, cola, kopi atau teh dengan gula, susu kental manis)	sweetened condensed milk)	[ ] 3. Satu kali per minggu<br>[ ] 4. 2-4 kali per minggu<br>[ ] 5. 5-6 kali per minggu<br>[ ] 6. 1 kali sehari<br>[ ] 7. Lebih dari satu kali sehari<br>[ ] 99. Menolak untuk menjawab	[ ] 4 2-4 times per week<br>[ ] 5 5-6 times per week<br>[ ] 6 Once a day<br>[ ] 7 More than once a day<br>[ ] 99 Refuse to answer
D19	Makanan olahan atau ultraproses (ikan kaleng, sosis, sayuran kaleng, makanan beku, dll.)	Processed or ultra-processed foods (canned fish, sausages, canned vegetables, frozen food, etc.)	[ ] 1. Tidak pernah atau kurang dari satu kali per bulan<br>[ ] 2. 1-3 kali per bulan<br>[ ] 3. Satu kali per minggu<br>[ ] 4. 2-4 kali per minggu<br>[ ] 5. 5-6 kali per minggu<br>[ ] 6. 1 kali sehari<br>[ ] 7. Lebih dari satu kali sehari<br>[ ] 99. Menolak untuk menjawab	[ ] 1 Never or less than once per month<br>[ ] 2 1-3 times per month<br>[ ] 3 Once a week<br>[ ] 4 2-4 times per week<br>[ ] 5 5-6 times per week<br>[ ] 6 Once a day<br>[ ] 7 More than once a day<br>[ ] 99 Refuse to answer
D20	Makanan penutup (misalnya, es krim, kue, permen, kue kering, ketan, makanan manis lainnya)	Dessert (e.g., ice cream, cake, candy, cookies, sticky rice with mango, other sweets)	[ ] 1. Tidak pernah atau kurang dari satu kali per bulan<br>[ ] 2. 1-3 kali per bulan<br>[ ] 3. Satu kali per minggu<br>[ ] 4. 2-4 kali per minggu<br>[ ] 5. 5-6 kali per minggu<br>[ ] 6. 1 kali sehari<br>[ ] 7. Lebih dari satu kali sehari<br>[ ] 99. Menolak untuk menjawab	[ ] 1 Never or less than once per month<br>[ ] 2 1-3 times per month<br>[ ] 3 Once a week<br>[ ] 4 2-4 times per week<br>[ ] 5 5-6 times per week<br>[ ] 6 Once a day<br>[ ] 7 More than once a day<br>[ ] 99 Refuse to answer

No	Pertanyaan	Question	Jawaban (Silahkan dicentang)	Answer (Please choose)
D21	Daging atau ikan yang digoreng (ikan goreng, ayam goreng, daging sapi goreng)	Deep-fried meat or fish (fried, fish, fried chicken, fried beef)	[ ] 1. Tidak pernah atau kurang dari satu kali per bulan<br>[ ] 2. 1-3 kali per bulan<br>[ ] 3. Satu kali per minggu<br>[ ] 4. 2-4 kali per minggu<br>[ ] 5. 5-6 kali per minggu<br>[ ] 6. 1 kali sehari<br>[ ] 7. Lebih dari satu kali sehari<br>[ ] 99. Menolak untuk menjawab	[ ] 1 Never or less than once per month<br>[ ] 2 1-3 times per month<br>[ ] 3 Once a week<br>[ ] 4 2-4 times per week<br>[ ] 5 5-6 times per week<br>[ ] 6 Once a day<br>[ ] 7 More than once a day<br>[ ] 99 Refuse to answer
