# Supplementary material for: Association between receiving the Aksi Bergizi Social Behavioral Change Communication (SBCC) intervention and dietary habits among secondary school students in Padang, Indonesia
Source: PLoS One. 2025 Sep 5;20(9):e0331312. doi: 10.1371/journal.pone.0331312 (PMC12412942; doi:10.1371/journal.pone.0331312)
Supplement: S1 Table — Supplementary Table 1 – Frequency of consuming selected food items among participants with dietary patterns identified in factor analysis (n = 506 students). (DOCX) [file pone.0331312.s007.docx]

# Supplementary Table 1. Frequency of consuming selected food items among participants with dietary patterns identified in factor analysis (n=506 students)

| **Food item** | **Dietary Pattern 1 ("High Protein & Processed Foods")** | **Dietary Pattern 2 ("Snacks & Sugary Drinks")** | **Dietary Pattern 3**  **("Healthier Diet")** |
| --- | --- | --- | --- |
| **Rice (white rice)** |  |  |  |
| Never or less than once per month | 0% | 0% | 0% |
| 1-3 times per month | 0% | 0% | 0% |
| Once a week | 0% | 0% | 0% |
| 2-4 times per week | 0.8% | 0% | 0% |
| 5-6 times per week | 0.8% | 0% | 0% |
| Once a day | 1.6% | 1.6% | 0.7% |
| More than once a day | 96.7% | 98.4% | 99.3% |
| **Refined wheat products (white bread, noodles)** |  |  |  |
| Never or less than once per month | 0.8% | 0% | 0% |
| 1-3 times per month | 0% | 1.6% | 3.7% |
| Once a week | 22.1% | 11.0% | 20.6% |
| 2-4 times per week | 71.3% | 80.3% | 69.1% |
| 5-6 times per week | 4.9% | 6.3% | 5.9% |
| Once a day | 0.8% | 0.8% | 0.7% |
| More than once a day | 0% | 0% | 0% |
| **Coarse grain (brown rice)** |  |  |  |
| Never or less than once per month | 98.4% | 97.6% | 97.8% |
| 1-3 times per month | 0.8% | 2.4% | 2.2% |
| Once a week | 0.8% | 0% | 0% |
| 2-4 times per week | 0% | 0% | 0% |
| 5-6 times per week | 0% | 0% | 0% |
| Once a day | 0% | 0% | 0% |
| More than once a day | 0% | 0% | 0% |
| **Whole grain wheat products (e.g., brown bread, whole wheat noodles)** |  |  |  |
| Never or less than once per month | 65.6% | 70.1% | 89.7% |
| 1-3 times per month | 13.9% | 16.5% | 7.4% |
| Once a week | 10.7% | 3.1% | 1.5% |
| 2-4 times per week | 9.8% | 9.4% | 1.5% |
| 5-6 times per week | 0% | 0% | 0% |
| Once a day | 0% | 0% | 0% |
| More than once a day | 0% | 0.8% | 0% |
| **Tubers (cassava, taro, white yams, white potato)** |  |  |  |
| Never or less than once per month | 1.6% | 0% | 0% |
| 1-3 times per month | 0.8% | 0.8% | 2.2% |
| Once a week | 11.5% | 3.9% | 1.5% |
| 2-4 times per week | 82.0% | 84.3% | 89.0% |
| 5-6 times per week | 4.1% | 11.0% | 7.4% |
| Once a day | 0% | 0% | 0% |
| More than once a day | 0% | 0% | 0% |
| **Meat (beef, mutton)** |  |  |  |
| Never or less than once per month | 0% | 0% | 0% |
| 1-3 times per month | 9.0% | 65.4% | 77.9% |
| Once a week | 70.5% | 22.0% | 18.4% |
| 2-4 times per week | 17.2% | 12.6% | 3.7% |
| 5-6 times per week | 0% | 0% | 0% |
| Once a day | 3.3% | 0% | 0% |
| More than once a day | 0% | 0% | 0% |
| **Poultry (duck, chicken)** |  |  |  |
| Never or less than once per month | 0% | 0% | 0% |
| 1-3 times per month | 3.3% | 17.3% | 12.5% |
| Once a week | 51.6% | 22.0% | 47.1% |
| 2-4 times per week | 39.3% | 56.7% | 35.3% |
| 5-6 times per week | 0% | 0% | 0% |
| Once a day | 4.9% | 3.9% | 4.4% |
| More than once a day | 0.8% | 0% | 0.7% |
| **Fish (raw, grilled, soup, not deep-fried)** |  |  |  |
| Never or less than once per month | 0% | 9.4% | 6.6% |
| 1-3 times per month | 2.5% | 50.4% | 41.9% |
| Once a week | 55.7% | 28.3% | 42.6% |
| 2-4 times per week | 32.8% | 10.2% | 2.9% |
| 5-6 times per week | 0% | 0% | 0% |
| Once a day | 6.6% | 0% | 5.9% |
| More than once a day | 2.5% | 1.6% | 0% |
| **Fresh seafood (e.g., clams, prawns, crabs, octopus)** |  |  |  |
| Never or less than once per month | 0.8% | 44.9% | 28.7% |
| 1-3 times per month | 11.5% | 39.4% | 58.8% |
| Once a week | 65.6% | 9.4% | 9.6% |
| 2-4 times per week | 13.9% | 6.3% | 0% |
| 5-6 times per week | 0% | 0% | 0% |
| Once a day | 8.2% | 0% | 2.9% |
| More than once a day | 0% | 0% | 0% |
| **Eggs** |  |  |  |
| Never or less than once per month | 0% | 0% | 0% |
| 1-3 times per month | 0% | 0% | 0.7% |
| Once a week | 0.8% | 0.8% | 1.5% |
| 2-4 times per week | 90.2% | 92.1% | 93.4% |
| 5-6 times per week | 5.7% | 7.1% | 4.4% |
| Once a day | 0% | 0% | 0% |
| More than once a day | 3.3% | 0% | 0% |
| **Leafy green vegetables (e.g., Chinese cabbage, long bean, kale, spinach, yu choy, cucumber)** |  |  |  |
| Never or less than once per month | 4.1% | 0% | 0% |
| 1-3 times per month | 0% | 0% | 0% |
| Once a week | 2.5% | 0.8% | 0% |
| 2-4 times per week | 3.3% | 0% | 0% |
| 5-6 times per week | 0% | 0% | 0% |
| Once a day | 6.6% | 34.6% | 11.8% |
| More than once a day | 83.6% | 64.6% | 88.2% |
| **Yellow or orange vegetables (e.g., pumpkin, sweet potatoes, carrots, ripened papaya)** |  |  |  |
| Never or less than once per month | 0% | 0% | 0% |
| 1-3 times per month | 0% | 0.8% | 0% |
| Once a week | 0% | 3.1% | 0% |
| 2-4 times per week | 86.1% | 79.5% | 80.1% |
| 5-6 times per week | 0% | 3.9% | 4.4% |
| Once a day | 10.7% | 9.4% | 13.2% |
| More than once a day | 3.3% | 3.1% | 2.2% |
| **Soybean products (e.g., tofu, *tempe*)** |  |  |  |
| Never or less than once per month | 4.1% | 0% | 0% |
| 1-3 times per month | 0% | 0% | 0% |
| Once a week | 9.0% | 0% | 0% |
| 2-4 times per week | 77.5% | 68.5% | 54.4% |
| 5-6 times per week | 7.4% | 29.9% | 43.4% |
| Once a day | 0% | 0% | 0.7% |
| More than once a day | 1.6% | 1.6% | 1.5% |
| **Preserved vegetables (e.g., canned pickled vegetables)** |  |  |  |
| Never or less than once per month | 93.4% | 91.3% | 97.8% |
| 1-3 times per month | 4.1% | 4.7% | 2.2% |
| Once a week | 0% | 0% | 0% |
| 2-4 times per week | 2.5% | 3.9% | 0% |
| 5-6 times per week | 0% | 0% | 0% |
| Once a day | 0% | 0% | 0% |
| More than once a day | 0% | 0% | 0% |
| **Fresh fruits** |  |  |  |
| Never or less than once per month | 2.5% | 3.9% | 0% |
| 1-3 times per month | 0% | 0% | 0% |
| Once a week | 4.9% | 21.3% | 11.0% |
| 2-4 times per week | 74.6% | 59.1% | 67.6% |
| 5-6 times per week | 1.6% | 5.5% | 4.4% |
| Once a day | 11.5% | 5.5% | 9.6% |
| More than once a day | 0% | 4.7% | 7.4% |
| **Dairy products (fresh milk, powdered milk, boxed milk)** |  |  |  |
| Never or less than once per month | 38.5% | 50.4% | 72.1% |
| 1-3 times per month | 0% | 1.6% | 3.7% |
| Once a week | 6.6% | 0.8% | 0% |
| 2-4 times per week | 4.1% | 12.6% | 5.9% |
| 5-6 times per week | 4.9% | 4.7% | 4.4% |
| Once a day | 44.3% | 29.1% | 13.2% |
| More than once a day | 1.6% | 0.8% | 0.7% |
| **Packaged snacks (e.g., corn puffs, Lay potato chips)** |  |  |  |
| Never or less than once per month | 3.3% | 3.9% | 1.5% |
| 1-3 times per month | 4.1% | 0% | 30.9% |
| Once a week | 39.3% | 3.9% | 31.6% |
| 2-4 times per week | 49.2% | 85.8% | 31.6% |
| 5-6 times per week | 0.8% | 0.8% | 0% |
| Once a day | 1.6% | 3.9% | 4.4% |
| More than once a day | 1.6% | 1.6% | 0% |
| **Unpackaged snacks (donuts, french fries, roti, etc.)** |  |  |  |
| Never or less than once per month | 0% | 0% | 0% |
| 1-3 times per month | 6.6% | 0% | 47.8% |
| Once a week | 45.1% | 3.9% | 19.9% |
| 2-4 times per week | 44.3% | 78.0% | 31.6% |
| 5-6 times per week | 1.6% | 11.0% | 0% |
| Once a day | 0.8% | 5.5% | 0.7% |
| More than once a day | 1.6% | 1.6% | 0% |
| **Sweetened drinks or condiments (soy milk, soft drinks, coke, coffee or tea with sugar, sweetened condensed milk)** |  |  |  |
| Never or less than once per month | 0% | 5.5% | 5.1% |
| 1-3 times per month | 6.6% | 3.9% | 38.2% |
| Once a week | 19.7% | 11.0% | 8.8% |
| 2-4 times per week | 71.3% | 70.1% | 41.9% |
| 5-6 times per week | 0% | 2.4% | 3.7% |
| Once a day | 1.6% | 6.3% | 2.2% |
| More than once a day | 0.8% | 0.8% | 0% |
| **Processed or ultra-processed foods (canned fish, sausages, canned vegetables, frozen food, etc.)** |  |  |  |
| Never or less than once per month | 0.8% | 13.4% | 22.1% |
| 1-3 times per month | 32.8% | 50.4% | 66.9% |
| Once a week | 41.8% | 15.7% | 5.1% |
| 2-4 times per week | 23.8% | 19.7% | 5.1% |
| 5-6 times per week | 0% | 0% | 0% |
| Once a day | 0.8% | 0.8% | 0.7% |
| More than once a day | 0% | 0% | 0% |
| **Dessert (e.g., ice cream, cake, candy, cookies, other sweets)** |  |  |  |
| Never or less than once per month | 0% | 0% | 0% |
| 1-3 times per month | 14.8% | 49.6% | 74.3% |
| Once a week | 45.9% | 23.6% | 5.1% |
| 2-4 times per week | 36.1% | 25.2% | 18.4% |
| 5-6 times per week | 0% | 0% | 0% |
| Once a day | 3.3% | 1.6% | 2.2% |
| More than once a day | 0% | 0% | 0% |
| **Deep-fried meat or fish (fried, fish, fried chicken, fried beef)** |  |  |  |
| Never or less than once per month | 0% | 0% | 0% |
| 1-3 times per month | 0% | 0% | 1.5% |
| Once a week | 15.6% | 0.8% | 1.5% |
| 2-4 times per week | 68.0% | 52.8% | 72.8% |
| 5-6 times per week | 16.4% | 46.5% | 24.3% |
| Once a day | 0% | 0% | 0% |
| More than once a day | 0% | 0% | 0% |
