# Supplementary material for: Association between receiving the Aksi Bergizi Social Behavioral Change Communication (SBCC) intervention and dietary habits among secondary school students in Padang, Indonesia
Source: PLoS One. 2025 Sep 5;20(9):e0331312. doi: 10.1371/journal.pone.0331312 (PMC12412942; doi:10.1371/journal.pone.0331312)
Supplement: Attachment — Submitted filename: PONE-D-24-31641_reviewer - Word.docx [file pone.0331312.s008.docx]

PLOS ONE

**Association between receiving the Aksi Bergizi Social Behavioral Change Communication (SBCC) intervention and dietary habits among secondary school students in Padang, Indonesia**

--Manuscript Draft--

| **Manuscript Number:** | PONE-D-24-31641 |
| --- | --- |
| **Article Type:** | Research Article |
| **Full Title:** | Association between receiving the Aksi Bergizi Social Behavioral Change Communication (SBCC) intervention and dietary habits among secondary school students in Padang, Indonesia |
| **Short Title:** | Social Behavioral Change Communication Intervention and Dietary Habits among Indonesian Students |
| **Corresponding Author:** | Wit Wichaidit  Prince of Songkla University Faculty of Medicine Hat Yai, Songkhla THAILAND |
| **Keywords:** | adolescents; Self-efficacy; Dietary habits; Nutrition promotion |
| **Abstract:** | Background: The Government of Indonesia and UNICEF introduced the Aksi Bergizi Social Behavioral Change Communication (SBCC) intervention to promote healthy dietary behaviors among adolescents. However, no systematic assessment of the Program’s effect has been made. The objectives of this study are: 1) to assess the extent to which exposure to the Aksi Bergizi Program is associated with dietary behaviors among secondary school students, and; 2) to assess mediation of the mentioned association by dietary self-efficacy.  Methods: We conducted a school-based cross-sectional study in Padang Municipality, Indonesia. We collected data from 253 students attending Aksi Bergizi target schools, and 253 students from non-target schools, using self-administered questionnaire with a food frequency questionnaire (FFQ) section. We analyzed data using descriptive statistics with analysis of mediation by self-efficacy in dietary consumption.  Results: We identified two distinct dietary patterns among the students: one characterized by poultry and snacks, and the other by fish and fruit. Students in the Aksi Bergizi target schools were significantly more likely than students in the non-target schools to have the Poultry and Snacks dietary pattern (57.3% vs. 41.5%, Adjusted OR  = 1.67; 95% CI = 1.08, 2.59), and also more likely to have the Fish and Fruits dietary pattern, although the difference was not statistically significant (32.0% vs. 26.9%, Adjusted OR = 1.19; 95% CI = 0.78, 1.81). Regarding mediation, students in the Aksi Bergizi target schools had significantly different dietary self-efficacy from students in the non-target schools (p<0.001), but other associations were not statistically significant.  Conclusion: The findings of this study have implications for stakeholders in adolescent health. However, limitations regarding the cross-sectional design, social desirability, and limited generalizability should be considered in the interpretation of the study findings. |
| **Order of Authors:** | Ricvan Dana Nindrea |
|  | Sawitri Assanangkornchai |
|  | Masrul Muchtar |
|  | Virasakdi Chongsuvivatwong |
|  | Wit Wichaidit |
| **Additional Information:** |  |
| **Question** | **Response** |
| **Financial Disclosure**  Enter a financial disclosure statement that | The author(s) received no specific funding for this work. |

*Powered by Editorial Manager® and ProduXion Manager® from Aries Systems Corporation*

| describes the sources of funding for the work included in this submission. Review the [submission guidelines](http://journals.plos.org/plosone/s/submission-guidelines#loc-financial-disclosure-statement) for detailed requirements. View published research articles from [*PLOS ONE*](https://journals.plos.org/plosone/) for specific examples.  This statement is required for submission and **will appear in the published article** if the submission is accepted. Please make sure it is accurate. |  |
| --- | --- |
| **Funded studies**  Enter a statement with the following details:   - Initials of the authors who received each award - Grant numbers awarded to each author - The full name of each funder - URL of each funder website - Did the sponsors or funders play any role in the study design, data collection and analysis, decision to publish, or preparation of the manuscript? |  |
| Did you receive funding for this work? |  |
| **Competing Interests**  Use the instructions below to enter a competing interest statement for this submission. On behalf of all authors, disclose any [competing interests](http://journals.plos.org/plosone/s/competing-interests) that could be perceived to bias this work—acknowledging all financial support and any other relevant financial or non- financial competing interests.  This statement is required for submission and **will appear in the published article** if the submission is accepted. Please make sure it is accurate and that any funding sources listed in your Funding Information later in the submission form are also declared in your Financial Disclosure statement.  View published research articles from  [*PLOS ONE*](http://journals.plos.org/plosone/) for specific examples. | The authors have declared that no competing interests exist |

*Powered by Editorial Manager® and ProduXion Manager® from Aries Systems Corporation*

| **NO authors have competing interests**  Enter: *The authors have declared that no competing interests exist.*  **Authors with competing interests**  Enter competing interest details beginning with this statement:  *I have read the journal's policy and the authors of this manuscript have the following competing interests: [insert competing interests here]* |  |
| --- | --- |
| * typeset |  |
| **Ethics Statement**  Enter an ethics statement for this submission. This statement is required if the study involved:   - Human participants - Human specimens or tissue - Vertebrate animals or cephalopods - Vertebrate embryos or tissues - Field research   Write "N/A" if the submission does not require an ethics statement.  General guidance is provided below. Consult the [submission guidelines](http://journals.plos.org/plosone/s/submission-guidelines#loc-guidelines-for-specific-study-types) for detailed instructions. **Make sure that all information entered here is included in the Methods section of the manuscript.** | This study received ethical approval from the ethics committee of the Faculty of Medicine at Prince Songkla University (Approval No. REC.66-248-18-2). |

*Powered by Editorial Manager® and ProduXion Manager® from Aries Systems Corporation*

| **Format for specific study types**  **Human Subject Research (involving human participants and/or tissue)**   - Give the name of the institutional review board or ethics committee that approved the study - Include the approval number and/or a statement indicating approval of this research - Indicate the form of consent obtained (written/oral) or the reason that consent was not obtained (e.g. the data were analyzed anonymously)   **Animal Research (involving vertebrate animals, embryos or tissues)**   - Provide the name of the Institutional Animal Care and Use Committee (IACUC) or other relevant ethics board that reviewed the study protocol, and indicate whether they approved this research or granted a formal waiver of ethical approval - Include an approval number if one was obtained - If the study involved *non-human primates*, add *additional details* about animal welfare and steps taken to ameliorate suffering - If anesthesia, euthanasia, or any kind of animal sacrifice is part of the study, include briefly which substances and/or methods were applied   **Field Research**  Include the following details if this study involves the collection of plant, animal, or other materials from a natural setting:   - Field permit number - Name of the institution or relevant body that granted permission |  |
| --- | --- |
|  |  |
| **Data Availability**  Authors are required to make all data underlying the findings described fully available, without restriction, and from the time of publication. PLOS allows rare exceptions to address legal and ethical concerns. See the [PLOS Data Policy](http://journals.plos.org/plosone/s/data-availability) and [FAQ](http://journals.plos.org/plosone/s/data-availability#loc-faqs-for-data-policy) for detailed information. | No - some restrictions will apply |

*Powered by Editorial Manager® and ProduXion Manager® from Aries Systems Corporation*

| A Data Availability Statement describing where the data can be found is required at submission. Your answers to this question constitute the Data Availability Statement and **will be published in the article**, if accepted. |  |
| --- | --- |
| **Important:** Stating ‘data available on request from the author’ is not sufficient. If your data are only available upon request, select ‘No’ for the first question and explain your exceptional situation in the text box. |  |
| Do the authors confirm that all data underlying the findings described in their manuscript are fully available without restriction? |  |
| **Describe where the data may be found in full sentences. If you are copying our sample text, replace any instances of XXX with the appropriate details.**   - If the data are **held or will be held in a public repository**, include URLs, accession numbers or DOIs. If this information will only be available after acceptance, indicate this by ticking the box below. For example: *All XXX files are available from the XXX database (accession number(s) XXX, XXX.*). - If the data are all contained **within the manuscript and/or Supporting Information files**, enter the following: *All relevant data are within the manuscript and its Supporting Information files.* - If neither of these applies but you are able to provide **details of access elsewhere**, with or without limitations, please do so. For example:   *Data cannot be shared publicly because of [XXX]. Data are available from the XXX Institutional Data Access / Ethics Committee (contact via XXX) for researchers who meet the criteria for access to confidential data.*  *The data underlying the results presented in the study are available from (include the name of the third party* | The data underlying the results presented in thie study are available from the corresponding author upon reasonable request. |

*Powered by Editorial Manager® and ProduXion Manager® from Aries Systems Corporation*

| *and contact information or URL).*   - This text is appropriate if the data are owned by a third party and authors do not have permission to share the data.   * typeset |  |
| --- | --- |
| Additional data availability information: |  |

*Powered by Editorial Manager® and ProduXion Manager® from Aries Systems Corporation*

Manuscript with data collection date	[Click here to access/download;Manuscript;Main File 20240729_Revised.docx](https://www2.cloud.editorialmanager.com/pone/download.aspx?id=37065530&guid=e672c7f5-0ad9-4c26-a329-ac3c63fd59fc&scheme=1)

# Association between receiving the *Aksi Bergizi* Social Behavioral Change Communication (SBCC) intervention and dietary habits among secondary school students in Padang, Indonesia

Ricvan Dana Nindrea^1,2^, Sawitri Assanangkornchai^1^, Masrul Muchtar^3^, Virasakdi Chongsuvivatwong^1^, Wit Wichaidit^1*^

# Affiliations:

1) Department of Epidemiology, Faculty of Medicine, Prince of Songkla University. Hat Yai, Thailand
2) Department of Medicine, Faculty of Medicine, Universitas Negeri Padang. Bukittinggi, Indonesia
3) Department of Nutrition, Faculty of Medicine, Andalas University. Padang, Indonesia

# Corresponding Author Wit Wichaidit

Department of Epidemiology, Faculty of Medicine, Prince of Songkla University, 15 Karnjaravanich Rd., Hat Yai, Songkhla 90110, Thailand

Email: [wit.w@psu.ac.th](mailto:wit.w@psu.ac.th)

# ABSTRACT

**Background:** The Government of Indonesia and UNICEF introduced the *Aksi Bergizi* Social Behavioral Change Communication (SBCC) intervention to promote healthy dietary behaviors among adolescents. However, no systematic assessment of the Program’s effect has been made. The objectives of this study are: 1) to assess the extent to which exposure to the *Aksi Bergizi* Program is associated with dietary behaviors among secondary school students, and; 2) to assess mediation of the mentioned association by dietary self-efficacy.

**Methods:** We conducted a school-based cross-sectional study in Padang Municipality, Indonesia. We collected data from 253 students attending *Aksi Bergizi* target schools, and 253 students from non-target schools, using self-administered questionnaire with a food frequency questionnaire (FFQ) section. We analyzed data using descriptive statistics with analysis of mediation by self-efficacy in dietary consumption.

**Results:** We identified two distinct dietary patterns among the students: one characterized by poultry and snacks, and the other by fish and fruit. Students in the *Aksi Bergizi* target schools were significantly more likely than students in the non-target schools to have the Poultry and Snacks dietary pattern (57.3% vs. 41.5%, Adjusted OR = 1.67; 95% CI = 1.08, 2.59), and also more likely to have the Fish and Fruits dietary pattern, although the difference was not statistically significant (32.0% vs. 26.9%, Adjusted OR = 1.19; 95% CI = 0.78, 1.81). Regarding mediation, students in the *Aksi Bergizi* target schools had significantly different dietary self-efficacy from students in the non-target schools (p<0.001), but other associations were not statistically significant.

**Conclusion:** The findings of this study have implications for stakeholders in adolescent health. However, limitations regarding the cross-sectional design, social desirability, and limited generalizability should be considered in the interpretation of the study findings.

**Keywords:** Adolescents, Self-efficacy, Dietary habits, Nutrition promotion

# INTRODUCTION

Adolescents face a double burden in malnutrition [1]. The World Health Organization (WHO) estimated that 19% of adolescents worldwide were stunted, 8% were underweight, 9% had wasting, 11% were overweight and 9% were obese [2]. Malnutrition in adolescents is partly attributed to dietary habits [3,4]. A meta-analysis study found that 35% of adolescents aged 12- 17 years did not consume fruit at least 1 time/per day, 21% did not consume vegetables at least once per day, 43% consumed carbonated soft drinks at least once per day, and 46% consumed fast food at least once per week [4]. Unhealthy dietary habits during adolescence can elevate the risk of non-communicable diseases (NCDs) during adolescence [5] and adulthood [6].

Indonesia, a middle-income country where approximately 16 percent of the population are adolescents [7], also faces problems in adolescent malnutrition, including lack of nutrients, obesity, and anemia [8,9]. To promote healthier dietary behavior among adolescents, the Government of Indonesia and the United Nations Children’s Fund (UNICEF) have designed and implemented an intervention based on Social Behavioral Change Communication (SBCC)

[9] in selected secondary schools nationwide known as the *Aksi Bergizi* Nutrition Promotion Program [10-12]. Although *Aksi Bergizi* has been implemented since the middle of 2022, no assessment has been made regarding how much exposure to the Program’s activities was associated with dietary behaviors. Furthermore, as Social Behavioral Change Communication’s theory of change posits that changes occur through psychosocial drivers of the target behavior, we hypothesize that the effect of the intervention program on dietary behaviors is mediated by self-efficacy. Findings from such assessment can contribute empirical evidence regarding the program’s effectiveness. Thus, the findings have implications for stakeholders in adolescent health for guiding future decisions regarding the Program’s extension or further modification. The objectives of this study are: 1) to assess the extent to which exposure to the *Aksi Bergizi* Nutrition Promotion Program is associated with dietary

behaviors among secondary school students in Padang, Indonesia, and; 2) to assess the extent to which the mentioned association is mediated by self-efficacy of dietary behaviors.

# METHODS

**Study design and setting**

We conducted a school-based cross-sectional study in Padang Municipality, West Sumatra Province, Indonesia.

# Study participants and sample size calculation

The study participants were secondary school students aged 12-15 years in Padang Municipality, West Sumatra Province, Indonesia. For sample size calculation, we hypothesized that 59% of students in schools that received the *Aksi Bergizi* Program at fruit at least once per day (p1 = 0.59) compared to 42% among students in schools without the *Aksi Bergizi* Program (p2 = 0.42) [13,14]. At 95% level of confidence and 80% power, we calculated the sample size for comparison of two proportions at 253 students per group, thus a total of 506 students.

**Exposure: *Aksi Bergizi* nutrition promotion program**

The aim of the *Aksi Bergizi* nutrition promotion program was to provide health education to adolescents aged 12-18 of all genders in the target schools through a series of activities based on Social Behavioral Change Communication (SBCC). Program activities included nutritional education and promotion of healthy eating habits. The theory of change was that by increasing adolescents' knowledge and awareness of good nutrition and a healthy lifestyle, the adolescents would be more likely to adopt healthy eating patterns. The Program tasked either the teachers at the school or nutrition staff from a nearby primary healthcare center (*puskesmas*) to provide health education and organize activities on the first week of every month. Details regarding the Program’s intervention are available from a public source [10-12]. As the *Aksi Bergizi* Program was implemented in entire schools, we assumed that all students in schools that were the Program’s target had been exposed to the Program.

# Outcomes: Dietary habits

We used the food frequency questionnaire built based on the items listed in a previous study on dietary behaviors in Indonesia to measure dietary habits among students in the participating schools [12]. The FFQ section consisted of 22 questions, focusing on food items that are localized or particular to Indonesia, with the following possible answer choices: never or less than once per month, 1-3 times per month, once a week, 2-4 times per week, 5-6 times per week, once a day, and more than once a day. We converted answers on the FFQ to binary responses (i.e., whether the participant ate a given food item more than once per week vs. once per week or fewer).

# Mediator: Self-efficacy

The mediator variable was self-efficacy. We measured self-efficacy using the questions “I am confident that I can limit my consumption of sugary drinks (e.g., soda, energy drinks) to one per week or less” and “I am confident that I can resist the temptation to engage in unhealthy foods (e.g., excessive snacking)” [12].

# Study instrument

Our study instrument was a self-administered paper and pen questionnaire. We designed the questionnaire in English and Bahasa Indonesia by adapting existing instruments [11,12]. We conducted a pilot-test of the study instrument in Solok Regency, which is one of the regencies/cities in West Sumatra Province. Further revision of the questionnaire was carried out through an iterative process. We also assessed the validity and reliability of the instruments during this period. These variables were assessed using a Likert scale. The questions we used underwent validity and reliability testing, resulting in a Cronbach's alpha value of >0.7. The final questionnaire contained 3 sections and took approximately 20 minutes to complete.

# Sampling methodology

The secondary school database was obtained from the Padang Municipality Education Office, West Sumatra Province, Indonesia. The sampling technique used in this study was multistage stratified clustered sampling. In the first stage, the secondary school database was obtained from the Padang Municipality Education Office, West Sumatra Province, Indonesia. A stratum was created based on secondary schools being targeted and non-targeted for the *Aksi Bergizi* nutrition program. Two schools were then randomly selected from each group. There were four secondary schools (all public) that were targeted for the *Aksi Bergizi* nutrition program and 39 secondary schools that were not targeted for the program. Two secondary schools were recruited from each group. In each school, we performed stratified random sampling of classrooms to select two classrooms per grade level using the list of classrooms provided by the school. To avoid any potential repercussions, we hereby refrain from disclosing the names of the schools.

# Data collection

We scheduled an appointment with the selected secondary schools to determine a feasible time and date for data collection in the selected classrooms. We then asked the principal or the teacher in charge to introduce the investigators to the students in the classrooms. The investigators informed the students about the study and requested their verbal informed consent. We requested and obtained a waiver of written informed consent and parental consent from our Institutional Review Boards (IRBs) in order to help us reassume the participants of their confidentiality. The information and consent processes were conducted in a group setting within the selected classes, but we allowed for individual questions and answers during the recruitment process. After the students expressed verbal consent, we directly distributed the

study questionnaires to the students and asked them to start filling out the questionnaires. We also ensured that no teacher was present in the classroom at the time. At the end of the data collection period, the students placed their questionnaire in an opaque envelope provided by the investigators, and placed the envelope in a locked box in front of the classroom or at a designated location. Data collected started on 9 November 2023 and ended on 2 December 2023.

# Data management

We opened the secured box in a private location and performed data entry on the paper questionnaires using the KoboToolbox platform, which uploaded the entered data to a password-protected server. For each questionnaire, two investigators performed data entry separately, and the principal investigator (RDN) checked for discrepancies between the two versions with regard to the unique identification number (ID) and other values, checked the original questionnaire, and made corrections accordingly.

# Data analysis

We used descriptive statistics to describe the characteristics of the study participants. We conducted bivariate analysis using the Chi-square test and multivariate logistic regression analysis, adjusting for potential confounders based on demographic characteristics. We performed exploratory factor analyses to identify dietary patterns. We decided on the appropriate number of patterns based on the Scree plot and a consensus among the investigators who performed data analyses. We then used the 75th percentile ranking of each dietary pattern as an arbitrary cut-off point for having such dietary pattern or the otherwise, and cross-tabulate the presence of such dietary pattern with the binary consumption of each food item to validate factor analysis results. Structural equation modelling (SEM) was used to analyze the data. A

confirmatory factor analysis (CFA) was employed to test the measurement model of the hypothesized model. A p-value of <0.05 was considered statistically significant.

# Ethical Considerations

This study received ethical approval from the ethics committee of the Faculty of Medicine at Prince Songkla University (Approval No. REC.66-248-18-2).

# RESULTS

A total of 253 students from the *Aksi Bergizi* target schools and 253 students from the *Aksi Bergizi* non-target schools participated in our study. There were significant socio-demographic differences between participants target and non-target schools (*Table 1*). While no significant differences were found in sex, religion, and BMI distributions, notable variations were observed. Targeted schools had a higher proportion of 14-year-old participants, fathers with an associate's degree, and households earning 3-4 million IDR monthly. Conversely, non-target schools had a higher percentage of fathers working as laborers/manual workers, mothers working as housewives, and mothers with a junior high school education. Additionally, non- targeted schools had more participants citing family and/or friends as their primary source of health behavior information. Students in the *Aksi Bergizi* target schools were less likely to self- report self-efficacy in maintaining a healthy diet, such as limiting the consumption of sugary drinks and excessive snacking.

Prevalence of dietary habits among students exposed to the *Aksi Bergizi* program (i.e. students at target school) and students not exposed to the program (i.e. students at non-target school) are as shown in *Table 2*. The consumption of packaged snacks, poultry, dairy products, unpackaged snacks, fresh fruits, and soybean products differed between the two groups, with exposed students consuming them more frequently, more than once per week. However, non- program students consumed fish more frequently than program students, showing a significant difference in their consumption habits.

We performed exploratory factor analyses for the food items with dichotomized frequencies (once a week or less often vs. more than once per week), the Scree plot suggested that there were 2 factors (*Figure 1*). The first factor loaded on 58% of variations in food consumption frequency (proportion explained by PA1 = 0.58) and second factor loaded on the remaining 42% (proportion explained by PA2 = 0.42) (*Table 3*). Based on the high factor loadings

correlation patterns, we decided to name the first factor the “*Dietary Pattern 1: Poultry and Snacks*” diet, and the second factor the “*Dietary Pattern 2: Fish and Fruits*” diet. Students in the *Aksi Bergizi* target schools were significantly more likely than students in the non-target schools to have the Poultry and Snacks dietary pattern (57.3% vs. 41.5%, Adjusted OR = 1.67; 95% CI = 1.08, 2.59), and also more likely to have the Fish and Fruits dietary pattern, although the difference was not statistically significant (32.0% vs. 26.9%, Adjusted OR = 1.19; 95% CI

= 0.78, 1.81).

Mediation of the association between exposure to *Aksi Bergizi* and dietary habits are shown in *Figure 2*. In the mediated pathway, being in *Aksi Bergizi* target schools (i.e., exposure to the *Aksi Bergizi* program) was significantly associated with the level of self-efficacy (p<0.001). However, the level of self-efficacy was not significantly associated with dietary habits. The non-mediated pathway between exposure to the *Aksi Bergizi* program and dietary habits was also not statistically significant.

Validation of factor analysis results by cross-tabulation between having dietary patterns identified by factor analysis by frequency of consumption of individual food items is shown in *Supplementary Table 1*.

# DISCUSSION

In this school-based cross-sectional study, we described the prevalence of self-reported health behaviors among students exposed to the *Aksi Bergizi* program and students not exposed to the program. We identified two distinct dietary patterns among the students: a Poultry and Snacks pattern and a Fish and Fruit pattern. Both of which were more common at *Aksi Bergizi* target school, although the difference with regard to Poultry and Snacks pattern was the only one that was statistically significant. The findings of our study have implications for stakeholders in adolescent health and school health.

Previous studies have highlighted the importance of nutrition education programs in promoting healthy dietary habits among adolescents [15-17]. However, in this study, we measured dietary behaviors only with the Food Frequency Questionnaire (FFQ), which did not measure the food items’ composition. In other words, there was no data with regards to food quantity, safety, salt and sugar contents, all of which can impact health. In Padang, a typical meal often includes a staple carbohydrate such as rice accompanied by a variety of side dishes that include meat (fish, chicken, beef), vegetables, and *sambal* (chili paste) [18,19]. Rice often comprise more than 50% of daily caloric intake [20,21], and traditional Padang cuisine has liberal use of salt, sugar, and oil [21], as well as coconut milk. Thus, it is possible that there was excessive intake of salt, sugar, and fat independent of food frequency patterns [20,21]. Future studies should consider using other measures, such as the 24-hour food recall, to gain additional insights [19,20].

We found that students at *Aksi Bergizi* target schools were more likely to frequently consume poultry and snacks than students at non-target schools. These findings are consistent with other school-based interventions on adolescent nutrition, which also report high consumption of snack foods and poultry among students [22,23]. The findings suggested that the *Aksi Bergizi* program’s effects in the target schools might have been weaker than anticipated. Regarding the mediation analysis, *Aksi Bergizi* seemed to change self-efficacy (a key behavioral driver) as

intended by the principles of social behavioral change communication, but self-efficacy might not have been the proper determinant of dietary behaviors. Designing future programs to change dietary behaviors should take *Aksi Bergizi*’s intervention into consideration and consider shifting the behavioral drivers of focus as deemed appropriate.

The strength of this study lies in the self-administration of the study questionnaire, which helps to reduce social desirability bias, to an extent. However, a number of limitations should be considered in the interpretation of our study findings. Firstly, the cross-sectional design only allows for the description of variations in risky health behaviors at a single point in time. Our participants may engage in more or less risk behaviors during their life course. Secondly, with standard curriculum in health education, self-report of eating unhealthy foods could be regarded as not socially desirable. Thus, we could not rule out the influence of social desirability in our study findings. Lastly, we only collected data from schools in Padang Municipality, which limited the generalizability of our study findings.

# CONCLUSION

In this school-based cross-sectional study, we assessed the extent to which dietary behavior of students differed between those in schools that did and did not receive the *Aksi Bergizi* social behavioral change communication (SBCC) program. We found that students in *Aksi Bergizi* target schools were significantly more likely to have a poultry and snacks based dietary pattern than students in the non-target schools, but mediation of such association by self-efficacy was not statistically significant. The findings of this study have implications for stakeholders in adolescent health. However, limitations regarding the cross-sectional design, social desirability, and limited generalizability should be considered in the interpretation of the study findings.

# Acknowledgements

We wish to thank all study participants and study school teachers and staff for their valuable time and assistance throughout this study. We also wish to thank our research assistants and data entry staff for their tireless efforts. We would like to thank the Padang Municipality Education Office of the Republic of Indonesia for their support and permission during the conduct of this study. This study was part of RDN's thesis, which was completed as partial fulfillment of the requirements for a Master of Science (M.Sc) degree in Epidemiology at Prince of Songkla University, Hat Yai, Songkhla, Thailand. The research reported in this publication was supported by the TUYF scholarship.

# Conflict of Interest

Nil

# REFERENCES

1. Seferidi P, Hone T, Duran A, Bernabe-Ortiz A, Millett C. Global inequalities in the double burden of malnutrition and associations with globalisation: a multilevel analysis of Demographic and Health Surveys from 55 low-income and middle-income countries, 1992-2018. Lancet Glob Health. 2022;10:e482–90.
2. Popkin B, Corvalan C, Grummer-Strawn L. Dynamics of the double burden of malnutrition and the changing nutrition reality. Lancet. 2020;395:65–74.
3. Mulu Birru G, Eshete Tadesse S, Hassen Abate K, Mekonnen T, Genetu Chane M. Malnutrition in School-Going Adolescents in Dessie Town, South Wollo, Ethiopia. J Nutr Metab. 2021;2021:4898970.
4. Beal T, Morris S, Tumilowicz A. Global Patterns of Adolescent Fruit, Vegetable, Carbonated Soft Drink, and Fast-Food Consumption: A Meta-Analysis of Global School- Based Student Health Surveys. Food Nutr Bull. 2019;40:444–59.
5. Smith L, Butler L, Tully M, Jacob L, Barnett Y, López-Sánchez G. Hand-Washing Practices among Adolescents Aged 12-15 Years from 80 Countries. Int J Environ Res Public Health. 2020;18:138.
6. Song Y, Liu J, Zhao Y, Gong L, Chen Q, Jiang X, et al. Unhealthy lifestyles and clusters status among 3637 adolescents aged 11-23 years: a school-based cross-sectional study in China. BMC Public Health. 2023;23(1):1279.
7. PopulationPyramid. Indonesia 2022 [Internet]. 2022 [accessed 26 June 2024]. Available from: <https://www.populationpyramid.net/indonesia/2022/>.
8. Ministry of Health, National Institute of Health Research and Development. National report on basic health research - RISKESDAS. Jakarta: Badan Litbangkes; 2018.
9. Ministry of Health, National Institute of Health Research and Development. National report on basic health research - RISKESDAS. Jakarta: Badan Litbangkes; 2013.
10. Unicef. Adolescent nutrition programme Aksi Bergizi: from district pilot to national scale- up. Jakarta: Unicef; 2019.
11. Direktur Gizi Masyarakat Kementerian Kesehatan Republik Indonesia. *Buku panduan untuk siswa Aksi Bergizi* (Handbook of Aksi Bergizi for students). Jakarta: Kementerian Kesehatan Republik Indonesia; 2019.
12. Direktur Gizi Masyarakat Kementerian Kesehatan Republik Indonesia. *Buku panduan untuk fasilitator Aksi Bergizi* (Handbook of Aksi Bergizi for facilitators). Jakarta: Kementerian Kesehatan Republik Indonesia; 2019.
13. Davis E, Cullen K, Watson K, Konarik M, Radcliffe J. A fresh fruit and vegetable program improves high school students’ consumption of fresh produce. J Am Diet Assoc. 2009;109:1227–31.
14. Ndagire C, Muyonga J, Nakimbugwe D. Fruit and vegetable consumption, leisure‐time physical activity, and sedentary behavior among children and adolescent students in Uganda. Food Sci Nutr. 2019;7:599–607.
15. Chaudhary A, Sudzina F, Mikkelsen BE. Promoting Healthy Eating among Young People- A Review of the Evidence of the Impact of School-Based Interventions. Nutrients. 2020;12:2894.
16. Raut S, Kc D, Singh DR, Dhungana RR, Pradhan PMS, Sunuwar DR. Effect of nutrition education intervention on nutrition knowledge, attitude, and diet quality among school- going adolescents: a quasi-experimental study. BMC Nutr. 2024;10:35.
17. Hamulka J, Wadolowska L, Hoffmann M, Kowalkowska J, Gutkowska K. Effect of an Education Program on Nutrition Knowledge, Attitudes toward Nutrition, Diet Quality, Lifestyle, and Body Composition in Polish Teenagers. The ABC of Healthy Eating Project: Design, Protocol, and Methodology. Nutrients. 2018;10:1439.
18. Lipoeto NI, Mmedsci, Agus Z, Oenzil F, Masrul M, Wattanapenpaiboon N. Contemporary Minangkabau food culture in West Sumatra, Indonesia. Asia Pac J Clin Nutr. 2001;10(1):10-6.
19. Lipoeto NI, Geok Lin K, Angeles-Agdeppa I. Food consumption patterns and nutrition transition in South-East Asia. Public Health Nutr. 2013;16:1637-43.
20. Lipoeto NI, Wattanapenpaiboon N, Malik A, Wahlqvist ML. Nutrition transition in west Sumatra, Indonesia. Asia Pac J Clin Nutr. 2004;13(3):312-6.
21. Gusnedi, Fahmida U, Djuwita R, Witjaksono F, Abdullah M. Food-based recommendations for Minangkabau women of reproductive age with dyslipidemia. Asia Pac J Clin Nutr. 2019;28(2):310-320.
22. Mihas C, Mariolis A, Manios Y, Naska A, Arapaki A, Mariolis-Sapsakos T, Tountas Y. Evaluation of a nutrition intervention in adolescents of an urban area in Greece: short- and long-term effects of the VYRONAS study. Public Health Nutr. 2010;13(5):712-9.
23. Ochoa-Avilés A, Verstraeten R, Huybregts L, Andrade S, Van Camp J, Donoso S, Ramírez PL, Lachat C, Maes L, Kolsteren P. A school-based intervention improved dietary intake outcomes and reduced waist circumference in adolescents: a cluster randomized controlled trial. Nutr J. 2017;16(1):79.

# Table 1. Characteristics of the study participants in *Aksi Bergizi* target and non-target schools (Frequency and %)

| **Characteristic** | ***Aksi Bergizi* target schools (n=253**  **students)** | ***Aksi Bergizi* non- target schools (n=253 students)** | **P-value** |
| --- | --- | --- | --- |
| **Sex** |  |  |  |
| Male | 106 (41.9%) | 111 (43.9%) | 0.719 |
| Female | 147 (58.1%) | 142 (56.1%) |  |
| **Age (years)** |  |  |  |
| 12 | 12 (4.7%) | 22 (8.7%) | **0.036** |
| 13 | 72 (28.5%) | 90 (35.6%) |  |
| 14 | 115 (45.5%) | 88 (34.8%) |  |
| 15 | 54 (21.3%) | 53 (20.9%) |  |
| **Ethnicity** |  |  |  |
| Minangnese | 146 (57.7%) | 167 (66.0%) | **<0.001** |
| Javanese | 35 (13.8%) | 57 (22.5%) |  |
| Bataknese | 8 (3.2%) | 0 (0%) |  |
| Sundanese | 5 (2.0%) | 0 (0%) |  |
| Others | 59 (23.3%) | 29 (11.5%) |  |
| **Father’s occupation** |  |  |  |
| Civil servant/ state enterprise | 54 (21.3%) | 74 (29.2%) | **<0.001** |
| Private sector employee | 92 (36.4%) | 79 (31.2%) |  |
| Small-scale vendors/ service  providers | 42 (16.6%) | 30 (11.9%) |  |
| Business owner/ entrepreneur | 26 (10.3%) | 10 (4.0%) |  |
| Laborer/ manual workers | 18 (7.1%) | 60 (23.7%) |  |
| Agriculture/ fishery | 19 (7.5%) | 0 (0%) |  |
| Independent professions (e.g.,  lawyers, architects) | 2 (0.8%) | 0 (0%) |  |
| **Father’s education** |  |  |  |
| Junior high school | 38 (15.0%) | 65 (25.7%) | **<0.001** |
| Senior high school | 86 (34.0%) | 93 (36.8%) |  |
| Vocational certificate | 1 (0.4%) | 0 (0%) |  |
| Associate’s degree | 70 (27.7%) | 26 (10.3%) |  |
| Bachelor’s degree | 51 (20.2%) | 69 (27.3%) |  |
| Higher than bachelor’s degree | 7 (2.8%) | 0 (0%) |  |
| **Mother’s occupation** |  |  |  |
| Housewife | 173 (68.4%) | 199 (78.7%) | **0.036** |
| Civil servant/ state enterprise | 15 (5.9%) | 12 (4.7%) |  |
| Private sector employee | 22 (8.7%) | 12 (4.7%) |  |
| Small-scale vendors/ service  providers | 39 (15.4%) | 30 (11.9%) |  |
| Business owner/ entrepreneur | 4 (1.6%) | 0 (0%) |  |
| **Mother’s education** |  |  |  |
| Primary school | 2 (0.8%) | 0 (0%) | **0.031** |
| Junior high school | 45 (17.8%) | 63 (24.9%) |  |

| **Characteristic** | ***Aksi Bergizi* target schools (n=253**  **students)** | ***Aksi Bergizi* non- target schools (n=253 students)** | **P-value** |
| --- | --- | --- | --- |
| Senior high school | 101 (39.9%) | 101 (39.9%) |  |
| Vocational certificate | 4 (1.6%) | 10 (4.0%) |  |
| Associate’s degree | 80 (31.6%) | 69 (27.3%) |  |
| Bachelor’s degree | 21 (8.3%) | 10 (4.0%) |  |
| **Household monthly income** |  |  |  |
| <1,000,000 IDR | 0 (0%) | 0 (0%) | **<0.001** |
| 1,000,000 to 2,000,000 IDR | 31 (12.3%) | 66 (26.1%) |  |
| 2,000,001 to 3,000,000 IDR | 61 (24.1%) | 77 (30.4%) |  |
| 3,000,001 to 4,000,000 IDR | 95 (37.5%) | 72 (28.5%) |  |
| 4,000,001 to 5,000,000 IDR | 47 (18.6%) | 33 (13.0%) |  |
| 5,000,001 to 6,000,000 IDR | 19 (7.5%) | 5 (2.0%) |  |
| **Religion** |  |  |  |
| Islam | 240 (94.9%) | 240 (94.9%) | 0.999 |
| Christianity | 13 (5.1%) | 13 (5.1%) |  |
| **Body mass index (BMI)** |  |  |  |
| Underweight (BMI <18.5 kg/m^2^) | 3 (1.2%) | 2 (0.8%) | 0.145 |
| Normal (BMI 18.5-22.9 kg/m^2^) | 245 (96.8%) | 238 (94.1%) |  |
| Overweight (23-24.9 kg/m^2^) | 5 (2.0%) | 13 (5.1%) |  |
| Obesity (≥25 kg/m^2^) | 0 (0%) | 0 (0%) |  |
| **Main sources of information about health behaviors** |  |  |  |
| Television advertisements | 63 (24.9%) | 41 (16.2%) | **0.003** |
| Family and/or friends | 131 (51.8%) | 169 (66.8%) |  |
| Social media platforms (e.g.  Facebook, X, Instagram, etc) | 59 (23.3%) | 43 (17.0%) |  |
| ***Dietary self-efficacy*** |  |  |  |
| **Agree or strongly agree:** I am confident that I can limit my consumption of sugary drinks (e.g., soda, energy drinks) to one  per week or less | 253 (100.0%) | 207 (81.8%) | **<0.001** |
| **Agree or strongly agree:** I am confident that I can resist the temptation to engage in unhealthy food (e.g., excessive  snacking) | 253 (100.0%) | 228 (90.1%) | **<0.001** |

**Table 2. Frequency of food consumption by item among students exposed to the *Aksi Bergizi* program and students not exposed to the program (frequency and %)**

| **Food item** | ***Aksi Bergizi* target schools (n=253**  **students)** | ***Aksi Bergizi* non-target schools (n=253**  **students)** | **P-value** | **Retain for factor analysis** |
| --- | --- | --- | --- | --- |
| **Rice (white rice)** |  |  | 0.999 | No |
| Once a week or less often | 1 (0.4%) | 0 (0%) |  |  |
| More than once per week | 252 (99.6%) | 253 (100.0%) |  |  |
| **Refined wheat products (white bread, noodles)** |  |  | 0.072 | **Yes** |
| Once a week or less often | 78 (30.8%) | 59 (23.3%) |  |  |
| More than once per week | 175 (69.2%) | 194 (76.7%) |  |  |
| **Coarse grain (brown rice)** |  |  | 0.999 | No |
| Once a week or less often | 253 (100.0%) | 253 (100.0%) |  |  |
| More than once per week | 1 (0.4%) | 0 (0%) |  |  |
| **Whole grain wheat products (e.g., brown bread, whole wheat noodles)** |  |  | 0.999 | No |
| Once a week or less often | 0 (0%) | 1 (0.4%) |  |  |
| More than once per week | 253 (100.0%) | 253 (100.0%) |  |  |
| **Tubers (cassava, taro, white yams, white potato)** |  |  | <0.001 | No |
| Once a week or less often | 21 (8.3%) | 0 (0%) |  |  |
| More than once per week | 232 (91.7%) | 253 (100.0%) |  |  |
| **Meet (beef, mutton)** |  |  | 0.124 | No |
| Once a week or less often | 0 (0%) | 4 (1.6%) |  |  |
| More than once per week | 253 (100.0%) | 249 (98.4%) |  |  |
| **Poultry (duck, chicken)** |  |  | **<0.001** | **Yes** |
| Once a week or less often | 151 (59.7%) | 202 (79.8%) |  |  |
| More than once per week | 102 (40.3%) | 51 (20.2%) |  |  |
| **Fish (raw, grilled, soup, not deep-fried)** |  |  | **<0.001** | **Yes** |
| Once a week or less often | 244 (96.4%) | 207 (81.8%) |  |  |
| More than once per week | 9 (3.6%) | 46 (18.2%) |  |  |
| **Fresh seafood (e.g., clams, prawns, crabs, octopus)** |  |  | 0.004 | No |
| Once a week or less often | 0 (0%) | 10 (4.0%) |  |  |
| More than once per week | 253 (100.0%) | 243 (96.0%) |  |  |
| **Eggs** |  |  | 0.005 | No |
| Once a week or less often | 14 (5.5%) | 2 (0.8%) |  |  |
| More than once per week | 239 (94.5%) | 251 (99.2%) |  |  |
| **Leafy green vegetables (e.g., Chinese cabbage,** |  |  | 0.075 | No |

| **Food item** | ***Aksi Bergizi* target schools (n=253**  **students)** | ***Aksi Bergizi* non-target schools (n=253**  **students)** | **P-value** | **Retain for factor analysis** |
| --- | --- | --- | --- | --- |
| **long bean, kale, spinach, yu choy, cucumber)** |  |  |  |  |
| Once a week or less often | 249 (98.4%) | 241 (95.3%) |  |  |
| More than once per week | 4 (1.6%) | 12 (4.7%) |  |  |
| **Yellow or orange vegetables (e.g., pumpkin, sweet potatoes, carrots, ripened papaya)** |  |  | 0.068 | No |
| Once a week or less often | 18 (7.1%) | 8 (3.2%) |  |  |
| More than once per week | 235 (92.9%) | 245 (96.8%) |  |  |
| **Soybean products (e.g., tofu, *tempe*)** |  |  | **0.003** | **Yes** |
| Once a week or less often | 9 (3.6%) | 27 (10.7%) |  |  |
| More than once per week | 244 (96.4%) | 226 (89.3%) | 999 |  |
| **Preserved vegetables (e.g., canned pickled vegetables)** |  |  | 0. | No |
| Once a week or less often | 253 (100.0%) | 253 (100.0%) |  |  |
| More than once per week | 1 (0.4%) | 0 (0%) |  |  |
| **Fresh fruits** |  |  | **0.005** | **Yes** |
| Once a week or less often | 40 (15.8%) | 67 (26.5%) |  |  |
| More than once per week | 213 (84.2%) | 186 (73.5%) |  |  |
| **Dairy products (fresh milk, powdered milk, boxed milk)** |  |  | **<0.001** | **Yes** |
| Once a week or less often | 174 (68.8%) | 218 (86.2%) |  |  |
| More than once per week | 79 (31.2%) | 35 (13.8%) |  |  |
| **Packaged snacks (e.g., corn puffs, Lay potato chips)** |  |  | **<0.001** | **Yes** |
| Once a week or less often | 125 (49.4%) | 180 (71.1%) |  |  |
| More than once per week | 128 (50.6%) | 73 (28.9%) |  |  |
| **Unpackaged snacks**  **(donuts, french fries, roti, etc.)** |  |  | **0.002** | **Yes** |
| Once a week or less often | 141 (55.7%) | 175 (69.2%) |  |  |
| More than once per week | 112 (44.3%) | 78 (30.8%) |  |  |
| **Sweetened drinks or condiments (soy milk, soft drinks, coke, coffee or tea with sugar, sweetened condensed milk)** |  |  | 0.005 | No |
| Once a week or less often | 14 (5.5%) | 2 (0.8%) |  |  |

| **Food item** | ***Aksi Bergizi* target schools (n=253**  **students)** | ***Aksi Bergizi* non-target schools (n=253**  **students)** | **P-value** | **Retain for factor analysis** |
| --- | --- | --- | --- | --- |
| More than once per week | 239 (94.5%) | 251 (99.2%) |  |  |
| **Processed or ultra- processed foods (canned fish, sausages, canned vegetables, frozen food, etc.)** |  |  | 0.075 | No |
| Once a week or less often | 249 (98.4%) | 241 (95.3%) |  |  |
| More than once per week | 4 (1.6%) | 12 (4.7%) |  |  |
| **Dessert (e.g., ice cream,**  **cake, candy, cookies, other sweets)** |  |  | 0.124 | No |
| Once a week or less often | 4 (1.6%) | 0 (0%) |  |  |
| More than once per week | 249 (98.4%) | 253 (100.0%) |  |  |
| **Deep-fried meat or fish (fried, fish, fried chicken, fried beef)** |  |  | 0.143 | No |
| Once a week or less often | 8 (3.2%) | 16 (6.3%) |  |  |
| More than once per week | 245 (96.8%) | 237 (93.7%) |  |  |

# Table 3. Factor loading for exploratory factor analysis of food consumption patterns among the study participants (n=506 students)

| **Food item** | **PA1** | **PA2** |
| --- | --- | --- |
| Refined wheat products (white  bread, noodles) | 0.31 | 0.30 |
| Poultry (duck, chicken) | 0.89* | 0.24 |
| Fish (raw, grilled, soup, not deep- fried) | 0.04 | 0.81* |
| Soybean products (e.g., tofu) | 0.04 | 0.21 |
| Fresh fruits | 0.11 | 0.74* |
| Dairy products (fresh milk,  powdered milk, boxed milk) | 0.16 | 0.17 |
| Packaged snacks (e.g., corn puffs,  Lay potato chips) | 0.88* | 0.30 |
| Unpackaged snacks (donuts, french  fries, roti, etc.) | 0.76* | 0.24 |
| **SS loadings** | 1.50 | 1.10 |
| **Proportion Var** | 0.21 | 0.16 |
| **Cumulative Var** | 0.21 | 0.37 |
| **Proportion Explained** | 0.58 | 0.42 |
| **Cumulative Proportion** | 0.58 | 1.00 |

**Denotes the variable being able to explain variations beyond the threshold (rho > 0.40 or rho < -0.40)*

# Table 4. Cross-tabulate dietary patterns found among students exposed to the *Aksi Bergizi* program and students not exposed to the program

| **Dietary patterns** | **Pattern not present** | **Pattern present** | **Unadjusted OR (95% CI)** | **Adjusted OR (95% CI)*** |
| --- | --- | --- | --- | --- |
| ***Dietary Pattern 1: Poultry and Snacks*** |  |  |  |  |
| *Aksi Bergizi* non-target  schools (n=253) | 148 (58.5%) | 105 (41.5% | *Reference* | *Reference* |
| *Aksi Bergizi* target schools  (n=253) | 108 (42.7%) | 145 (57.3%) | 1.89 (1.33-  2.69) | 1.67 (1.08-  2.59) |
| ***Dietary Pattern 2: Fish and Fruits*** |  |  |  |  |
| *Aksi Bergizi* non-target  schools (n=253) | 185 (73.1%) | 68 (26.9%) | *Reference* | *Reference* |
| *Aksi Bergizi* target schools  (n=253) | 172 (68.0%) | 81 (32.0%) | 1.28 (0.87-  1.88) | 1.19 (0.78-  1.81) |

*Adjusted for sex, age, ethnicity, father's occupation, father's education, mother's occupation, mother's education, household monthly income, main source of information about health behaviors, and dietary self-efficacy

Figure 1	[Click here to access/download;Figure;Figure 1.docx](https://www2.cloud.editorialmanager.com/pone/download.aspx?id=37056653&guid=d515a302-cd17-4da4-8bad-81273839ec66&scheme=1)

# Figure 1. Scree plot of dietary patterns classification among the participants

Figure 2	[Click here to access/download;Figure;Figure 2.docx](https://www2.cloud.editorialmanager.com/pone/download.aspx?id=37056654&guid=03190a49-b15a-4152-ac01-225a61356308&scheme=1)

# Figure 2. Structural equation models of mediation of the association between exposure to

***Aksi Bergizi* and dietary habits**

***Legend:***

*SE_20 = Self-Efficacy in Dietary Behaviors; Agree or strongly agree: I am confident that I can limit my consumption of sugary drinks (e.g., soda, energy drinks) to one per week or less*

*SE_21 = Self-Efficacy in Dietary Behaviors; Agree or strongly agree: I am confident that I can resist the temptation to engage in unhealthy behaviors (e.g., excessive snacking, skipping exercise)*

Supplementary Table 1

[Click here to access/download](https://www2.cloud.editorialmanager.com/pone/download.aspx?id=37056655&guid=b73e6521-675f-4eac-b8f2-20d2d7cd5b7c&scheme=1)

[**Supporting Information** Suppl Table 1.docx](https://www2.cloud.editorialmanager.com/pone/download.aspx?id=37056655&guid=b73e6521-675f-4eac-b8f2-20d2d7cd5b7c&scheme=1)
