# Supplementary material for: Association between receiving the Aksi Bergizi Social Behavioral Change Communication (SBCC) intervention and dietary habits among secondary school students in Padang, Indonesia
Source: PLoS One. 2025 Sep 5;20(9):e0331312. doi: 10.1371/journal.pone.0331312 (PMC12412942; doi:10.1371/journal.pone.0331312)
Supplement: Attachment — Submitted filename: R1_Editors and reviewers comments.docx [file pone.0331312.s009.docx]

**Response to the Editor’s Suggestions**

Please ensure that your manuscript meets PLOS ONE's style requirements, including those for file naming. The PLOS ONE style templates can be found at

<https://journals.plos.org/plosone/s/file?id=wjVg/PLOSOne_formatting_sample_main_body.pdf> and

[https://journals.plos.org/plosone/s/file?id=ba62/PLOSOne_formatting_sample_title_authors_affiliations.pdf](https://track.editorialmanager.com/CL0/https:%2F%2Fjournals.plos.org%2Fplosone%2Fs%2Ffile%3Fid=ba62%2FPLOSOne_formatting_sample_title_authors_affiliations.pdf/1/010f01943e4f5ab2-5c08a956-2dd5-4c2f-ba90-9bf46a9b6a9f-000000/vJOSkGV3YblyInqOl-1yJfjNly2C4dAJ-XrAdq1sa-M=192)

Please include a complete copy of PLOS’ questionnaire on inclusivity in global research in your revised manuscript. Our policy for research in this area aims to improve transparency in the reporting of research performed outside of researchers’ own country or community. The policy applies to researchers who have travelled to a different country to conduct research, research with Indigenous populations or their lands, and research on cultural artefacts. The questionnaire can also be requested at the journal’s discretion for any other submissions, even if these conditions are not met.  Please find more information on the policy and a link to download a blank copy of the questionnaire here: <https://journals.plos.org/plosone/s/best-practices-in-research-reporting>. Please upload a completed version of your questionnaire as Supporting Information when you resubmit your manuscript.

COMMENT

In the online submission form, you indicated that [The data underlying the results presented in this study are available from the corresponding author upon reasonable request.]. All PLOS journals now require all data underlying the findings described in their manuscript to be freely available to other researchers, either 1. In a public repository, 2. Within the manuscript itself, or 3. Uploaded as supplementary information.This policy applies to all data except where public deposition would breach compliance with the protocol approved by your research ethics board. If your data cannot be made publicly available for ethical or legal reasons (e.g., public availability would compromise patient privacy), please explain your reasons on resubmission and your exemption request will be escalated for approval.

RESPONSE

We thank the editor for the comment and notification. We will upload the minimal data set necessary to replicate the findings of this study as Supplementary Information along with our codes when we resubmit the manuscript along with the data dictionary. We will also include the English-language translation of our study questionnaire as an additional Supplementary Information section material.

COMMENT

Please include a separate caption for each figure in your manuscript.

RESPONSE

We thank the editor for the comment and have included a separate caption for each figure accordingly

**REVIEWER 1**

COMMENT

The questions above promotes the academic standard of the article and meeting the World academic standard

RESPONSE

We thank the reviewer for the comments in the Word document and have tried to address them on a point-by-point basis below

*Responses to Reviewer 1's comments in the Word document*

**Abstract**

COMMENT

The abstract was poorly written, and Grammarly software is recommended for grammar editing

RESPONSE

We thank the reviewer for the comment and we apologize for the quality of the manuscript. We have used Grammarly to correct the errors accordingly. If the reviewer deems that grammatical mistakes remain in the revised manuscript, please kindly indicate the lines at which they are found in the next iteration and we will make our best efforts to correct them in the subsequent phases.

**INTRODUCTION**

COMMENT

Paragraph 1: Rewrite the sentence to capture the meaning of the paragraph, and use Grammarly for the grammar editing

RESPONSE

We thank the reviewer for the comment and we have use Grammarly to make corrections as per the reviewer's suggestions.

COMMENT

Paragraph 2: Use Grammarly for grammar editing

RESPONSE

We thank the reviewer for the comment and we have use Grammarly to make corrections as per the reviewer's suggestions.

**METHODS**

COMMENT

Study design and setting: Rewrite the sentence be formal writing

RESPONSE

We checked our sentence structure and vocabulary use with Grammarly and make revisions to the sub-section accordingly.

COMMENT

Study participants and sample size calculation: Rewrite the paragraph to explain the paragraph clearly and use Grammarly for grammar editing. Indicate the method used in calculating the sample size and the internal consistency of the instruments used

RESPONSE

We thank the reviewer for the comments. We have made the following changes to the latter half of the Study Participants and Sample Size Calculation sub-section as follows:

"Using the sample size calculation formula for comparison of two proportions and procedures for calculation using the R statistical environment assuming 95% level of confidence and 80% power, we obtained the sample size of 253 students per group, thus a total of 506 students."

COMMENT

Section (Exposure: Aksi Bergizi nutrition promotion program): Rewrite ‘’Exposure of Aksi Bergizi nutrition promotion program”; Difficult to express, rewrite to express its meaning; Use Grammarly software for grammar editing

RESPONSE

We thank the reviewer for the comment. The sub-section is meant to describe the exposure, i.e., the Aksi Bergizi nutrition promotion program. Thus, to avoid confusion, we wish to revise the sub-section header to "The *Aksi Bergizi* nutrition promotion program" with italics on the non-English terms. We have also used Grammarly to revise the manuscript accordingly. Changes made where deemed necessary.

COMMENT

Section (Outcomes: Dietary habits): Change to ‘Dietary habits outcome’; Rewrite the paragraph with formal writing and use Grammarly software for grammar editing

RESPONSE

We thank the reviewer for the comment. The sub-section is meant to describe the measurement of the outcome, i.e., dietary habits of the students in the Aksi Bergizi target and non-target schools. Therefore, we decided to revise the header of the sub-section to "Measurement of dietary habits". We have also used Grammarly to revise the manuscript accordingly. Changes made where deemed necessary.

COMMENT

Section (Mediator: Self-efficacy): Rewrite as ‘Mediator variable: Self-efficacy’; Change to formal writing

RESPONSE

We thank the reviewer for the comment. We decided to revise the header of the sub-section to "Measurement of the mediator (self-efficacy)". We have also used Grammarly to revise the manuscript accordingly. Changes made where deemed necessary.

COMMENT

Sampling methodology: Change to’ research design and sampling’; Rewrite to express the meaning of the paragraph

RESPONSE

We thank the reviewer for the suggestion. The sub-section is meant to describe the sampling procedures, and we have already described the study design at the beginning of the METHODS section. Thus, we wish to suggest an alternative header: "Sampling procedures". We have also revised the paragraph where deemed appropriate as per the reviewer's comments.

COMMENT

Data collection: This is not formal writing, express the paragraph in a formal writing; At this level the authors must be sure of the exact place to drop the questionnaire

RESPONSE

We thank the reviewer for the comments. To improve clarity, we have revised the second half of the sub-section as follows:

"At the end of the data collection period, the students placed their questionnaire in an opaque envelope provided by the investigators, and placed the envelope in a locked box in front of the classroom or at a location otherwise designated by the investigators."

We have also made changes to the other parts of the sub-section as deemed appropriate, including revisions according to Grammarly's suggestions

**RESULTS**

COMMENT

"Targeted schools had a higher proportion of 14-year-old participants, fathers with an associate's degree...": Table 1 shows that Father has higher Senior high school of 34.0%

RESPONSE

We thank the reviewer for the comment. Just to clarify, our narrations were meant to be a comparison of the characteristics between Aksi Bergizi target schools and non-target schools, and not a description of the participants in the target schoosl only. We have re-checked the findings in Table 1 and wish to hereby respectfully request that we retain the existing descriptions to align with the presented numbers in Table 1.

COMMENT

"Conversely, non-target schools had a higher percentage of fathers working as laborers/manual workers, mothers working as housewives, and mothers with a junior high school education.": This is wrong, the higher percentage was Civil servant/ state enterprise (29.2%); This is wrong, the higher percentage is Senior high school (39.9%)

RESPONSE

We thank the reviewer for the comments. We wish to offer the same response as that to the previous comment

COMMENT

Second paragraph: Rewrite to clearly explain the sentence; This sentence is difficult to express, kindly rewrite it to explain the sentence better; This sentence is confusing, are non-program students the same thing as ‘ non-target schools’?

RESPONSE

We thank the reviewer for the comment. We have tried to make changes accordingly with the help of Grammarly

COMMENT

Third paragraph: Always write with official writing, this no good for academic writing ; Rewrite the discussion to clearly explain the tables and figure 1

RESPONSE

We thank the reviewer for the suggestions and we have made revisions accordingly.

**DISCUSSION**

COMMENT

The discussion was poorly written without citing an article of similar findings. Rewrite the discussion section to better explain the finding results

RESPONSE

We thank the reviewer for the comment and we have added more citations where we deemed appropriate.

COMMENT

"Future studies should consider using other measures, such as the 24-hour food recall, to gain additional insights [19,20]." This should be discussed under recommendation for further studies

RESPONSE

We thank the reviewer for the comment. The authors have deliberated and felt that the remark addressed the preceding issue that would be more appropriate where it is already located, thus we wish to request that we keep the statement as it appears.

COMMENT

Paragraph three: The article concept is good but poorly expressed, kindly rewrite to clearly discuss the result

RESPONSE

We thank the reviewer for the comment. We have revised the manuscript accordingly and made changes where deemed appropriate.

COMMENT

Paragraph four: This sentence is under limitation of the stud

RESPONSE

We thank the reviewer for the comment. To clarify, the paragraph is meant to communicate the strengths and limitations of the study, with a focus on the latter. Therefore, we wish to keep the content as it appears, but we also welcome more specific suggestions in the next iteration

**CONCLUSION**

COMMENT

Rewrite the paragraph to clearly explain the paragraph

RESPONSE

We thank the reviewer for the comment. We have revised the manuscript accordingly and made changes where deemed appropriate.

**Reviewer 2**

1. Dietary pattern analysis typically relies on intake data of various food groups. It may not be appropriate to utilize binary variables (more than once per week vs. once per week or fewer) for such analysis, as this approach may result in a loss of information. Furthermore, the authors have emerged 22 food groups into only eight, which raises concerns regarding the data resolution

RESPONSE

We thank the reviewer for the comment. After deliberations, we agree that using binary variables put us at potential loss of information. We decided that a better (albeit imperfect) approach would be for us to use the reported frequency of consumption as it appeared in the questionnaire, and to use all 22 food groups. We also note that the food frequency questionnaire output values were not meant to be analyzed as discrete numbers, and that errors in the finding regarding patterns of consumption could have resulted. Changes made extensively to the RESULTS section (Tables 2 thru 4). We have identified three dietary patterns instead of two patterns. We have also made changes to the DISCUSSION section according to the revised results.

2. Do the two dietary patterns identified in the manuscript accurately reflect the local dietary habits of Indonesia? The authors may suggest that the fish and fruit dietary pattern is favorable; however, it is essential to determine whether this finding aligns with the original objectives of the Aksi Bergizi Program intervention.

RESPONSE

We thank the reviewer for the comment. To answer the reviewer's question, the investigators deemed that the patterns of dietary habits in the Poultry and Snacks group are consistent with the preferences of adolescents in the region. However, the Fish and Fruits dietary pattern was also consistent with the behaviors that the Aksi Bergizi program was trying to promote.

Because we re-analyzed the data, the findings in the RESULTS section in this iteration of the manuscript differ substantially from those in the previous version. In the DISCUSSION section, we have added remarks regarding consistency between the promoted narrative and behaviors in the *Aksi Bergizi* program and the observed dietary patterns accordingly.

3. The self-efficacy assessment presented in the article appears overly simplistic, consisting of only two questions. It may be beneficial to employ a more comprehensive scale for this evaluation.

RESPONSE

We thank the reviewer for the comment. Unfortunately, we cannot change the evaluation scale as we have already finished our data collection activity. However, we acknowledge and appreciate the call to explain the measurement of self-efficacy in a more complete manner and have made changes to the METHODS section accordingly.

4. Before constructing Structural Equation Models (SEMs), a clear theoretical framework or supporting data usually should be established. At least, a comparative analysis of self-efficacy levels between the two groups is needed.

RESPONSE

We thank the reviewer for the comment and we have added a short overview of the literature and presented the hypothesis underlying our Structural Equation Model (SEM) in the INTRODUCTION section accordingly.

5. The conclusion of the article appears ambiguous. While the SEM analysis indicates that the Aksi Bergizi Program was associated with self-efficacy, it was not associated with dietary habits. Moreover, the pathway between self-efficacy and dietary habits was also found to be insignificant.

RESPONSE

We thank the reviewer for the comment. When we made the first draft of the manuscript, we noticed the lack of statistical significance in the association between the Aksi Bergizi Program and dietary habits, and between self-efficacy and dietary habits. We acknowledged that our hypothesis was not supported by our findings, but we did not make remarks or discussions extensively. Additional details and remarks added to the DISCUSSION section, including a suggestion that other behavioral drivers should be considered.
