# Supplementary material for: Association between receiving the Aksi Bergizi Social Behavioral Change Communication (SBCC) intervention and dietary habits among secondary school students in Padang, Indonesia
Source: PLoS One. 2025 Sep 5;20(9):e0331312. doi: 10.1371/journal.pone.0331312 (PMC12412942; doi:10.1371/journal.pone.0331312)
Supplement: Attachment — Submitted filename: R2_Editors and reviewers comments.docx [file pone.0331312.s010.docx]

**Response to the Reviewers’ Comments**

**REVIEWER 1**

COMMENT

The authors have improved on the manuscript's corrections; however, before the manuscript's publication, the authors need to make the following corrections:

RESPONSE

We thank the reviewer for the comment on this iteration. We have made revisions accordingly, particularly by making the sentences more formal and moving the sentences in the DISCUSSION section to the Recommendation part of the CONCLUSION section

COMMENT

1. Do grammar editing for the manuscript because some of the sentences were informal

RESPONSE

We thank the reviewer for the comment. We have tried to make the sentences more formal accordingly.

COMMENT

2. The sentences in the red ink under the discussion section should move to the recommendation section.

RESPONSE

We have moved the sentences in the DISCUSSION section and added the following remarks to the Recommendation part of the CONCLUSION section:

"Based on the study findings and the associated issues, we have two main recommendations for future studies and health problems. Firstly, future studies should consider using other measures of dietary intake, such as the 24-hour food recall, to gain additional insights regarding adolescent dietary behaviors. Secondly, future public health programs targeting adolescent dietary behaviors should consider other key behavioral drivers in addition to self-efficacy as the mediator in the behavior change process."

**REVIEWER 2**

(No Response)

**REVIEWER 3**

COMMENT

Thank you for your submission. Your study addresses an important topic—the association between a school-based SBCC intervention and dietary habits among adolescents in Indonesia. The subject is timely and valuable, particularly in LMICs where adolescent nutrition remains a pressing challenge. I appreciate your efforts in collecting primary data and incorporating structural equation modeling to explore mediation pathways.

That said, several methodological and conceptual aspects need further clarification and improvement to strengthen the manuscript:

RESPONSE

We thank the reviewer for the comments and we have tried to revise them accordingly.

COMMENT

The cross-sectional design limits causal interpretation. Please emphasize this more clearly in the abstract and conclusion.

RESPONSE

We thank the reviewer for the comment. We have revised the final sentence in the Conclusion part of the ABSTRACT section to the following:

"However, limitations regarding the cross-sectional design (which precludes the ability to make causal inference), social desirability, and limited generalizability should be considered in the interpretation of the study findings."

We have also included the following remarks in the CONCLUSION section:

"The limitations of the study, including the cross-sectional study design (which precludes the ability to make causal inference), the potential information biases, and the limited generalizability, should be considered as caveats in the interpretation of the study findings."

COMMENT

The binary classification of food frequency data reduces the granularity of your dietary behavior measurements. A justification for this decision is warranted, and limitations should be acknowledged more thoroughly.

RESPONSE

We thank the reviewer for the comment. We have added the following remarks to the DISCUSSION section:

"Furthermore, in our analyses, we dichotomized the responses to the food frequency data as the original answers were ordinal variables with unequal intervals between the categories. Including the responses as integers could have introduced errors during exploratory factor analysis. Thus, we decided to collapse the responses into binary classifications. Such a practice is common in factor analysis and could help to yield clear dietary patterns. However, one key limitation of this process is the loss of details, and the dichotomization as more than once per week vs. once per week or less also implies that there was no seasonality in the consumption of the food items. In the study area, families may consume meat only once per year during religious festivals, but do so in considerable quantity each day for up to one week (e.g., during Hari Raya Eid-Adha), yet the dichotomization presented in our study would not have captured this habit. Incorporating other measurement methods to capture these variations, such as the 24-hour food recall recorded throughout the year, could yield additional insights and enable more detailed measurements."

COMMENT

The self-efficacy scale includes only two items, which weakens the mediation analysis. While you mention this in the response to reviewers, the issue should be more critically discussed in the manuscript.

RESPONSE

We thank the reviewer for the comment. We have added the following remarks to the DISCUSSION section:

"The lack of association between self-efficacy and dietary habits in our SEM thus challenges our proposed theoretical model, and suggests the need to explore other potential mediators in the association between SBCC intervention and dietary behaviors."

We have added the following remarks to the CONCLUSION section:

"…future public health programs targeting adolescent dietary behaviors should consider other key behavioral drivers in addition to self-efficacy as the mediator in the behavior change process."

COMMENT

Your SEM results show that self-efficacy is not significantly associated with dietary habits. This challenges your proposed theoretical model and suggests the need to explore other potential mediators or behavioral determinants.

RESPONSE

We thank the reviewer for the comment. We have added the following remarks to the DISCUSSION section:

"The lack of association between self-efficacy and dietary habits in our SEM thus challenges our proposed theoretical model, and suggests the need to explore other potential mediators in the association between SBCC intervention and dietary behaviors."

We have also added the following remarks to the CONCLUSION section:

"...future public health programs targeting adolescent dietary behaviors should consider other key behavioral drivers in addition to self-efficacy as the mediator in the behavior change process."

COMMENT

The finding that students exposed to the Aksi Bergizi program were more likely to follow the "Snacks and Sugary Drinks" pattern is counterintuitive. Please consider offering more context or possible explanations in the discussion section.

RESPONSE

We thank the reviewer for the comment. We have added the following remarks to the DISCUSSION section accordingly:

"This unexpected change was not found in a similar study in another region of Indonesia. Another study showed that a school in Indonesia with on-site food service with a nutritionist had better nutrient adequacy among students than a school that had catering without a nutritionist. Thus, it is possible that the lack of a catering component with nutritionist involvement could explain for parts of the observed unhealthy dietary patterns in our study."

COMMENT

In addition, I recommend a final round of language editing to enhance academic clarity and consistency across the manuscript.

RESPONSE

We thank the reviewer for the suggestion. We have made our best attempt to edit our writing to improve clarity and consistency accordingly.
