# Supplementary material for: Association between receiving the Aksi Bergizi Social Behavioral Change Communication (SBCC) intervention and dietary habits among secondary school students in Padang, Indonesia
Source: PLoS One. 2025 Sep 5;20(9):e0331312. doi: 10.1371/journal.pone.0331312 (PMC12412942; doi:10.1371/journal.pone.0331312)
Supplement: Attachment — Submitted filename: R3_Editors and reviewers comments.docx [file pone.0331312.s011.docx]

**Response to the Reviewers’ Comments**

**JOURNAL REQUIREMENTS**

COMMENT

Please review your reference list to ensure that it is complete and correct. If you have cited papers that have been retracted, please include the rationale for doing so in the manuscript text, or remove these references and replace them with relevant current references. Any changes to the reference list should be mentioned in the rebuttal letter that accompanies your revised manuscript. If you need to cite a retracted article, indicate the article’s retracted status in the References list and also include a citation and full reference for the retraction notice.

RESPONSE

We thank the Editor for communicating the requirement. To the best of our knowledge, there are no retracted papers in our citations.

**REVIEWER 1**

COMMENT

(Attachment)

RESPONSE

The attachment with Reviewer 1’s comments appeared to be from the first round of review (dated 19 November 2024 and submitted to the journal on 20 November 2024), and the commented document appeared to be our original submission. We have made changes to the manuscript based on these comments when we submitted the first revision (R1). Thus, we decided to refrain from addressing the comments in Reviewer 1’s attached document

**REVIEWER 2**

COMMENT

(No Response)

RESPONSE

(No Response)

**REVIEWER 3**

COMMENT

The authors have carefully addressed all the comments and suggestions raised during the previous round of review. The revised manuscript demonstrates significant improvements in clarity, methodology, and presentation. All major and minor concerns have been adequately resolved, and no further revisions are necessary from my perspective.

Therefore, I find the current version of the manuscript suitable for publication in its present form.

RESPONSE

We thank the reviewer for the comment, and confirm keeping the manuscript as it appears.
